# Chalcogen Bond as a Factor Stabilizing Ligand Conformation in the Binding Pocket of Carbonic Anhydrase IX Receptor Mimic

**DOI:** 10.3390/ijms232213701

**Published:** 2022-11-08

**Authors:** Kamil Wojtkowiak, Mariusz Michalczyk, Wiktor Zierkiewicz, Aneta Jezierska, Jarosław J. Panek

**Affiliations:** 1 Faculty of Chemistry, University of Wrocław, ul. F. Joliot-Curie 14, 50-383 Wrocław, Poland; 2 Faculty of Chemistry, Wrocław University of Science and Technology, Wybrzeże Wyspiańskiego 27, 50-370 Wrocław, Poland

**Keywords:** Carbonic Anhydrase IX mimic, acetazolamide, non-covalent interactions, binding pocket, METD, DFT, SAPT, CPMD, PIMD

## Abstract

It is postulated that the overexpression of Carbonic Anhydrase isozyme IX in some cancers contributes to the acidification of the extracellular matrix. It was proved that this promotes the growth and metastasis of the tumor. These observations have made Carbonic Anhydrase IX an attractive drug target. In the light of the findings and importance of the glycoprotein in the cancer treatment, we have employed quantum–chemical approaches to study non-covalent interactions in the binding pocket. As a ligand, the acetazolamide (AZM) molecule was chosen, being known as a potential inhibitor exhibiting anticancer properties. First-Principles Molecular Dynamics was performed to study the chalcogen and other non-covalent interactions in the AZM ligand and its complexes with amino acids forming the binding site. Based on Density Functional Theory (DFT) and post-Hartree–Fock methods, the metric and electronic structure parameters were described. The Non-Covalent Interaction (NCI) index and Atoms in Molecules (AIM) methods were applied for qualitative/quantitative analyses of the non-covalent interactions. Finally, the AZM–binding pocket interaction energy decomposition was carried out. Chalcogen bonding in the AZM molecule is an important factor stabilizing the preferred conformation. Free energy mapping via metadynamics and Path Integral molecular dynamics confirmed the significance of the chalcogen bond in structuring the conformational flexibility of the systems. The developed models are useful in the design of new inhibitors with desired pharmacological properties.

## 1. Introduction

Non-covalent interactions are one of the most important forces allowing biomolecules to effectively form intricate structures [1,2]. The contemporary understanding of every kind of these interactions in chemistry is founded on the Lewis acid/base concept proposed in 1916 [3,4]. The Lewis base (LB) is an entity associated with an area of a large excess of electronic density and, on the other hand, Lewis acid (LA) is a term describing molecules or atoms associated with depletion of electron density acting like electrophiles [5]. It is important to note that the non-covalent interaction could be described as an interaction between LA and LB. For atoms involved in multipole–multipole interactions, the properties such as: polarizability, electronegativity and, directly related with them, anisotropic distribution of electron density are key to obtain an insight into the investigated systems [6,7].

One of the most famous examples is the hydrogen-bonding (HB) patterns in α-helical secondary structures of proteins, which were described by Pauling back in 1951 [8], or base-stacking and base-pairing contributions to the stability of the DNA double helix [9]. In addition, hydrogen bonds can play important roles in the catalytic processes or molecular recognition [10,11,12,13,14].

Interestingly, even a single HB influences the whole structure of a biomolecule, and the most pronounced example of this is the photochromism control in fluorescent proteins—the planar conformation of the chromophore requires up to three HBs formation, whereas one HB cannot prevent the chromophore from isomerization and promotes photo-switching [15]. The chalcogen bond is one of several subclasses belonging to the σ-hole theory, which is based on the local reduction of electron density on the outermost fragment of a chalcogen atom which is involved in an intramolecular covalent bond with an electron-withdrawing group [16,17,18,19,20,21,22,23,24,25]. As the outcome, the positively charged area on this chalcogen atom can act as a Lewis acid.

Recently, different types of these interactions including chalcogen, pnicogen, tetrel, triel, aerogen as well as halogen bonds have been extensively studied [26,27,28,29,30,31,32,33]. In the chalcogen bond, the sulfur atom serves as an LA. The chalcogen bonds are as much directional and energetic as the halogen bonds—whereas both, halogen and chalcogen bonds, are remarkably more directional than the hydrogen bonds [34,35,36]. Surprisingly, chalcogen bonds are not so well studied as pnicogen bonds, despite their great importance in the protein tertiary structure stability and drug design, where the attractive intermolecular interactions between sulfur σ-holes and the LB, such as aromatic rings, oxygen or nitrogen atoms, are the most prevalent [37].

As it is the case for other non-covalent interactions, the chalcogen bond strength rises with the polarizability of the participating atom (thus, as the atomic number of an atom of the 16th group increases, the strength of the bond rises), the basicity of interacting atom and the polarization of LA induced by the backbone. Moreover, the characteristic features of strong chalcogen bonds are bond angles close to 180 degrees [5,36,38]. In addition, due to the relative similarity of atoms from the 16th group to the halogens, the chalcogen bonds are similar to the former in bond lengths, interaction energies, electron charge density distributions or Laplacian values [39]. The charge transfer, dispersion and electrostatics are the main contributions to the attractive nature of the chalcogen bond, whereas the dispersion forces are especially important for weak complexes [40].

**Figure 1:**
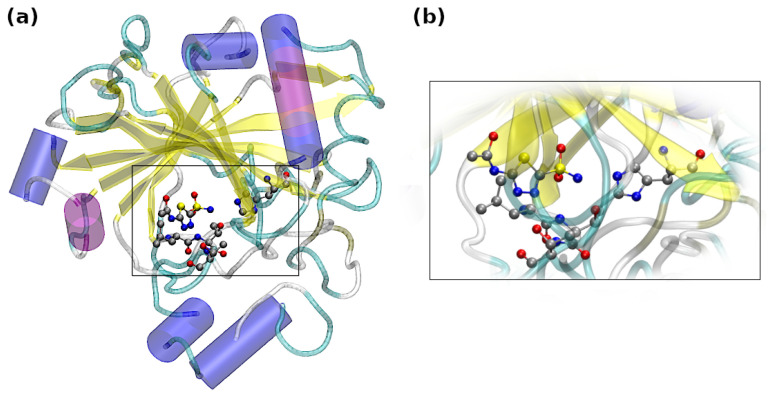
Visualization of the (**a**) ligand and the Carbonic Anhydrase IX mimic and (**b**) the magnified binding site of the Carbonic Anhydrase IX mimic. Cartoon representation was chosen for the protein, whereas the investigated amino acids and ligand were presented with usage of a ball-and-stick model, color coding: silver—carbon, yellow—sulfur, blue—nitrogen, red—oxygen.

**Figure 2:**
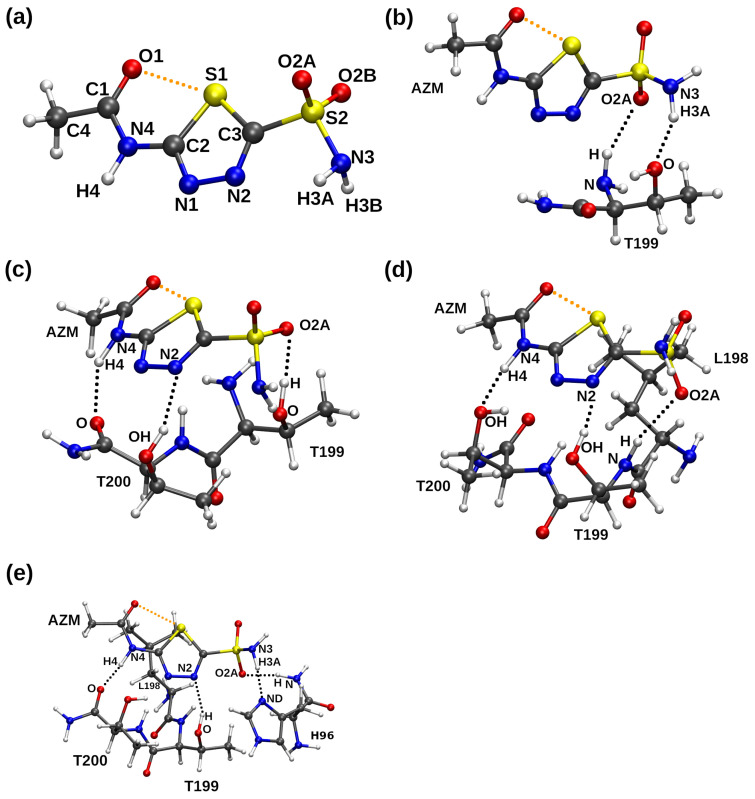
Molecular structure of (**a**) acetazolamine (AZM), and its complexes with CA IX mimic binding pocket (**b**) AZM-T, (**c**) AZM-TT, (**d**) AZM-TTL and (**e**) AZM-TTLH. The atoms coloring scheme is as follows: carbon—gray, oxygen—red, nitrogen—blue, sulfur—yellow, hydrogen—white. Hydrogen bonds are denoted as black dotted lines, while the chalcogen bonds are shown as orange dotted lines. The atoms numbering scheme used in the text is shown as well. The numbers of amino acids involved in the interactions with AZM molecules are depicted.

In order to study in detail the interactions between the ligand and protein with emphasis put on chalcogen and hydrogen bonds, the Carbonic Anhydrase (CA) IX mimic with an acetazolamide (AZM) ligand was chosen [41]. It is worth underlining that a significant homology between Carbonic Anhydrase isozymes was used to recreate the active site of CA IX. In its construction, as a basis was the Carbonic Anhydrase II crystal structure. The amino acids Ala65 and Asn67 have been replaced by Ser and Gln, respectively [41]. Carbonic Anhydrases are metalloenzymes dependent on zinc, and their function is to perform the reversible interconversion of CO_2_ and HCO_3_^−^. Additionally, the Carbonic Anhydrase IX is a membrane-embedded glycoprotein considered to be the marker of tumor hypoxia [42]. Recently, numerous studies suggest that the overexpression of Carbonic Anhydrase IX promotes the metastasis and the growth as well as the chemoresistance [43] of the tumor by the acidification of the extracellular matrix—therefore, as it is postulated, an inhibition of Carbonic Anhydrase IX protein can lead to the decreased invasiveness and to the cell death under the oxygen-deficient conditions [44,45]. Therefore, we have investigated in detail the competition/cooperativity of non-covalent interactions in the binding pocket of the Carbonic Anhydrase IX mimic with acetazolamide (AZM) ligand (the binding site and the whole protein structure are depicted in Figure 1). Acetazolamide has a pKa of 7.2, and its sulfonamide group can exist in –NH_2_ or –NH^–^ forms. In the current study, the neutral form was considered. Due to the scarcity of the chalcogen bond studies in the context of biologically active molecules and computational chemistry, in-depth knowledge of the interactions between the sulfur-containing molecules and the Carbonic Anhydrase IX binding site (see Figure 2) can facilitate a rational design of potential anticancer drugs. Furthermore, the approach applied in the current paper should shed new light onto non-covalent interactions as a whole, due to studying their behavior as the function of time and with respect to the temperature.

In silico studies of potential drugs can significantly reduce the cost of putting them on the market and can facilitate the comprehension of binding modes and structure–activity relationships that constitute the basis of their mechanisms of action. Thus, one can argue that the experimental work devoted to the development of the new biologically active substances should be intimately connected with the theoretical studies in order to obtain an optimal efficacy of the whole project [46]. While initial screening techniques of drug design strategies necessarily include fast high-throughput methods followed by docking, a rational in-depth design of bioactive molecules involves understanding their mechanisms of action. This can be achieved by analyzing interactions between the ligand and the host (receptor), and at this point, the importance of dynamical description arises. Effects such as allosteric regulation cannot be described without inspecting the details of protein dynamics. The “key and lock” paradigm of the substrate and enzyme binding site was modified by accepting the role of dynamical changes affecting both the ligand and the host. These facts prompted us to extend our investigations to encompass a detailed dynamical analysis of the conformational flexibility of the studied systems as well as a static description of the non-covalent interactions.

In this light, we have employed diverse theoretical chemistry approaches to develop time-evolution as well as static models to describe non-covalent interactions in the binding pocket and relations between them. First-Principle Molecular Dynamics (FPMD) methods, namely Car–Parrinello Molecular Dynamics (CPMD) [47], Path Integral Molecular Dynamics (PIMD) [48,49] and metadynamics (METD) [50] were employed to investigate the dynamical features of the AZM molecule and associated amino acids (derived from the binding pocket). Density Functional Theory (DFT) in its classical formulation was applied for geometric and electronic structure parameters. The Atoms in Molecules (AIM) [51,52,53] and Natural Bond Orbitals (NBO) [54,55] theories were employed to give a quantitative insight into electronic structure and topological parameters evolution of AZM upon the number of interacting amino acids from the Carbonic Anhydrase IX binding pocket. In order to understand the origin of stability of the studied complexes, the Non-Covalent Interaction (NCI) index [56], Symmetry-Adapted Perturbation Theory (SAPT) [57,58] and Energy Decomposition Analysis (EDA) [59] were applied. Finally, we would like to underline that to the best of our knowledge, it is the first and comprehensive study where the Carbonic Anhydrase IX receptor mimic binding pocket with an emphasis put on chalcogen bond and other non-covalent interactions have been investigated on the basis of FPMD approaches.

## 2. Results and Discussion

### 2.1. Metric and Spectroscopic Signatures of Non-Covalent Interactions

#### 2.1.1. Car–Parrinello Molecular Dynamics Description of the AZM ligand and Its Complexes with Amino Acids

Car–Parrinello Molecular Dynamics was employed to study metric and spectroscopic features of non-covalent interactions in the AZM ligand and its complexes with amino acids. We have analyzed the intra- and intermolecular chalcogen bonds as well as the network of intermolecular hydrogen bonds. The intramolecuar chalcogen bonds’ geometric parameters were derived from X-ray data [41], CPMD runs as well as static DFT, and they are presented in Table S1 (see Figure 2 for atoms numbering scheme). As it was found, the computed interatomic distance S1•••O1 in the AZM molecule is shorter (2.799 Å—averaged CPMD and 2.751 Å static DFT) than the experimental one (2.972 Å). The same tendency was maintained for the AZM complexes with amino acids (in addition, see Figure S1 in the Supplementary Material, where the time-evolution of S1•••O1 distance is graphically presented). A similar conclusion was made for the S1•••O2A and S1•••O2B chalcogen bonds. An interesting phenomenon was observed for the S1•••O2A and S1•••O2B chalcogen bonds during the CPMD simulations. The S2O2AO2BNH2 moiety rotated, and there was a “switch” between chalcogen bonds formed by S1•••O2A and S1•••O2B, respectively. It was observed for the AZM, AZM-T and AZM-TT complexes. An attachment of additional amino acids (AZM-TTL and AZM-TTLH cases) interacting with the AZM induced the lack of this phenomenon. An extended discussion with the graphical presentation (see Figures S2 and S3 for the metric parameters and Figure S4 for the spectroscopic analysis) of the results is presented in the Supplementary Material. The static DFT models were further developed to investigate the S1•••O1 chalcogen bond. The S1-C2-N4-C1 dihedral angle was rotated to provide potential energy profiles (see Figure S5 and Table S2). It was found that the energy barrier varies from 8.959 to 10.804 kcal/mol depending on the applied method. The lowest energy barrier value was obtained for the M05-D3(0)/aug-cc-pVDZ while the highest was obtained for the M05-2X-D3(0)/aug-cc-pVDZ levels of theory, respectively. The energy barrier value obtained by the MP2 method was 9.638 kcal/mol. In addition, the CCSD and CCSD(T) simulations were performed using the geometry of the AZM molecule obtained at the MP2/aug-cc-pVDZ level of theory. We have obtained an energy barrier of 9.726 kcal/mol and 9.575 kcal/mol, respectively. We can conclude that the provided benchmark confirmed that the meta-GGA functionals performed well for the investigated system.

#### 2.1.2. Nuclear Quantum Effects: Car-Parrinello vs. Path Integral Molecular Dynamics

**Figure 3:**
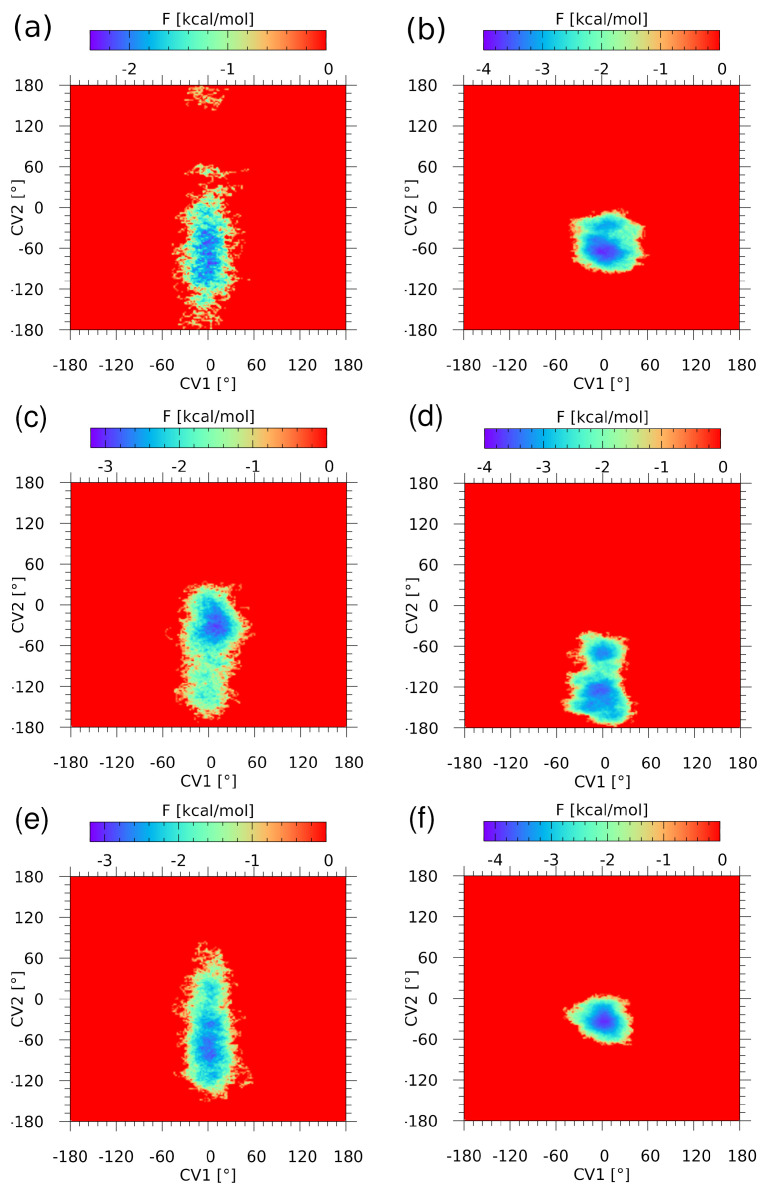
Free energy (F) surface reconstructed from (**a**) the CPMD run and (**b**) the PIMD run (with NQE inclusion) for the AZM molecule, (**c**) the CPMD run and (**d**) the PIMD run for the AZM-T system, (**e**) the CPMD run and (**f**) the PIMD run for the AZM-TT system. The collective variables are the following dihedral angles: CV1 is the S1-C2-N4-C1 dihedral, and CV2 is the S1-C3-S2-O2B dihedral (see Figure 2 for atoms numbering scheme).

Path Integral MD [48,60] calculations have been carried out to estimate the importance of the heavy atom skeleton quantization (nuclear quantum effects, NQE) for the molecular properties. The hydrogen-bonded systems can exhibit sensitivity to such quantization, as shown for the malonaldehyde proton transfer free energy profile [61]. However, we are not aware of such quantum treatment for systems with chalcogen bonds. The impact of NQE is visible primarily in the free energy profile for the relevant structural parameters. In the case of the isolated molecule, the selected parameters are two dihedral angles, S1-C2-N4-C1 (CV1) and S1-C3-S2-O2B (CV2), representing the conformation of the AZM molecule. The comparison of CPMD (classical nuclei) and PIMD (with NQE) free energy profiles for selected interaction models is presented in Figure 3. The difference between the CPMD and PIMD picture for the isolated AZM molecule (Figure 3a,b) is twofold. First, the CPMD free energy map, surprisingly, indicates larger conformational freedom of the molecule than its PIMD counterpart, especially with regard to the CV2 variable. This might result from the NQE strengthening of the intramolecular N3-H3A•••N2 contact. Moreover, the inclusion of NQE broadens slightly the CV1 profile and deepens the free energy well significantly. This indicates that the NQE in the heavy atom skeleton of the AZM molecule provides noticeable impact on the local minimum potential well. Further comparison of the free energy profiles for the binding site interaction models, AZM-T (Figure 3c,d) and AZM-TT (Figure 3e,f), leads to similar conclusions. For both the AZM-T and AZM-TT systems, the CPMD free energy landscape is similar to the case of the isolated AZM molecule, with large variations in the CV2 variable and very small deviations from 0 for the CV1 variable (directly describing the intramolecular chalcogen interaction within the AZM molecule). However, the intermolecular contacts described in the previous section lead to modifications in the exact location of the bottom of the free energy well. An inclusion of NQE leads to a dramatic restriction of the CV2 variability and broadening of the CV1 distribution. The clamping effect on the CV2 variable is not strongly associated with a particular model—the panels (b) and (f) of Figure 3 are similar—indicating the importance of intramolecular conformational stabilization via S1•••O1 chalcogen and N3-H3A•••N2 contacts. However, we have to notice that the thermal motions alone allow the system to explore only the regions relatively close to the bottom of the energy well; therefore, we have undertaken the metadynamics study to allow the structures’ escape from the local minima.

#### 2.1.3. Free Energy Landscapes Derived from CPMD-Based Metadynamics

**Figure 4:**
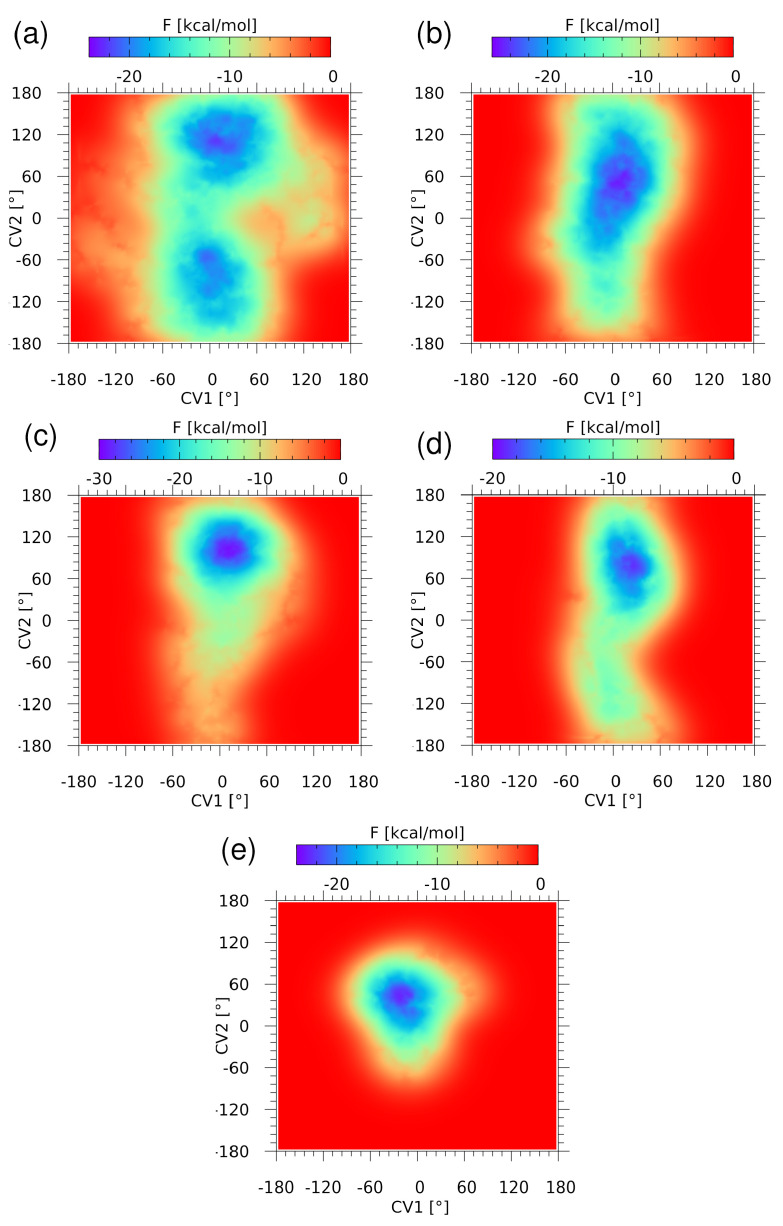
Free energy (F) surface reconstructed from the metadynamics run for (**a**) the AZM molecule, (**b**) the AZM-T, (**c**) the AZM-TT, (**d**) the AZM-TTL, and (**e**) the AZM-TTLH. The collective variables are the following dihedral angles: CV1 is the S1-C2-N4-C1 dihedral, and CV2 is the S1-C3-S2-O2B dihedral (see Figure 2 for atoms numbering scheme).

Metadynamics (METD) [50] allowed us to explore the free energy (F) landscape related to the non-covalent interactions. Two dihedral angles, S1-C2-N4-C1 (CV1) and S1-C3-S2-O2B (CV2), were used as collective variables (CV) in the CPMD-based METD description of the isolated AZM molecule and its complexes. In the case of the isolated AZM molecule, two deep minima of F have been found (see Figure 4a), but both of them correspond to the value of CV1 close to zero, maximizing the S1•••O1 chalcogen bond strength. The shortest path connecting these minima requires an uphill walk along the CV2 coordinate from −22 to −12 kcal/mol. On the other hand, the movement along the CV1 coordinate is much more costly, which can be attributed, at least partially, to the stabilizing intramolecular chalcogen bonding. Gradual entrapment of the AZM molecule inside the binding site models leads to strong restriction of the accessible conformational space (see Figure 4b–e). The CPMD runs described above in Section 2.1.1 indicate a growing complexity of the involved interactions, and this brings severe limitation to the variability range of the CV2 dihedral angle. First, the two minima coalesce into one broad minimum for AZM-T (see Figure 4b), which then narrows down significantly when more residues are added to the binding site model. The depth of the well is roughly constant, close to 22–24 kcal/mol, making the AZM ligand bound in place firmly. The CV1 dihedral angle, describing the S1•••O1 chalcogen bond, is less diverse in each of the complexes, underlining the importance of this contact for the preferred ligand conformation.

### 2.2. Electronic Structure Evidences of the Non-Covalent Network of Intra- and Intermolecular Interactions

**Figure 5:**
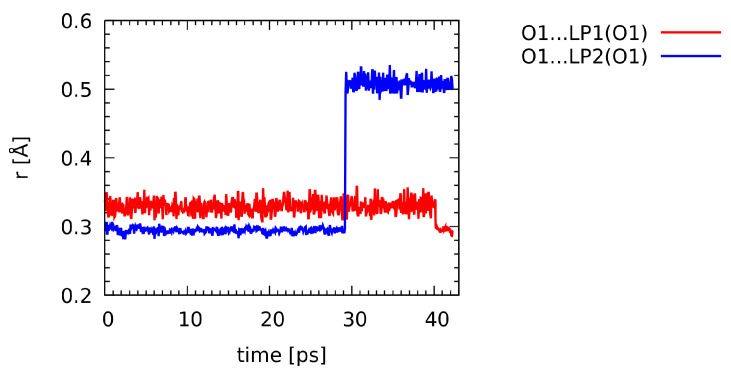
Distances between the O1 atom and its lone pairs centers (localized Wannier orbitals) along the CPMD trajectory. See Figure 2 for the atoms numbering scheme.

Wannier orbitals, corresponding to the transformation of the plane-wave molecular orbitals to the localized form, have been calculated at regular intervals along the CPMD trajectory. The S1•••O1 chalcogen bond involves primarily one of the lone pairs of the O1 oxygen atom. The distances between the O1 atom position and the centers of the lone pair orbitals for this atom were plotted along the CPMD trajectory—see Figure 5. The red line corresponds to the lone pair initially forming the chalcogen interaction. It can be seen that the distance is very stable, fluctuating around 0.34 Å, and it drops to 0.30 Å only after 40 ps of the simulation. The chalcogen bond at this point of the trajectory becomes weakened due to the internal conformational movement within the AZM molecule (change in the S1-C2-N4-C1 dihedral angle). The position of the Wannier center associated with the lone pair LP2(O1) exhibits more diverse behavior, which is also connected with the conformational changes of the molecule (at ca. 29 ps of the simulation time).

### 2.3. Non-Covalent Interactions Analysis Based on the NCI Index

**Figure 6:**
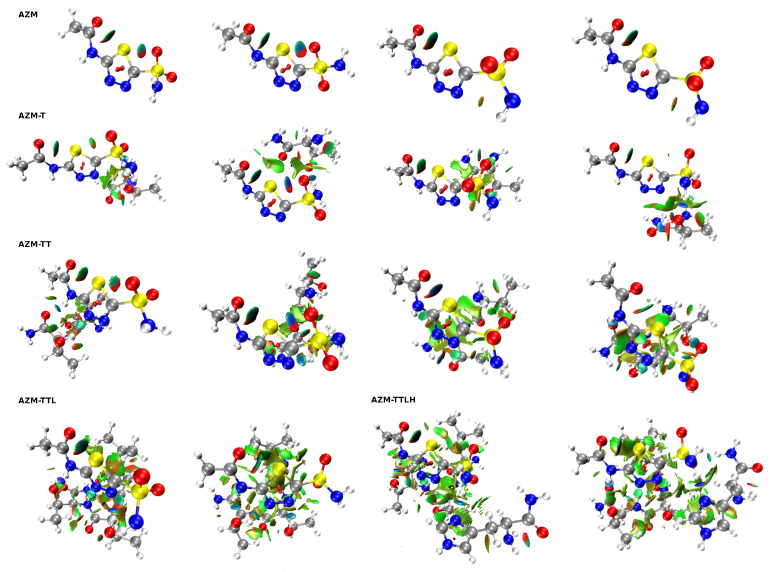
NCI diagrams of systems taken from the CPMD and optimized at the M06/aug-cc-pVDZ level of theory. Three types of geometries were taken from the course of the CPMD, namely: the structure with the smallest distance between S1 and O2A, the structure with the smallest distance for S1 and O2B, and the other structures, when the S1•••O2A and S1•••O2B distances were equal to each other. Diagrams of optimized structures are presented in the last column of every row (the exception is AZM-TTL, for which it is placed in the second column). In addition to optimized ones, in the case of AZM-TTL and AZM-TTLH, only geometries with values closest to the averaged distance were taken.

Non-Covalent Interaction (NCI) index analysis is a pictorial way of discussing electron density topology with special emphasis on the non-covalent interactions. In Figure 6, the overall structure of the NCIs present in the studied systems—optimized as well as the CPMD snapshots—is given. For ease of the initial discussion, the spatial distribution of the NCI regions for the optimized systems is also present in Figure S6 in the Supplementary Material, where black arrows point to the green areas corresponding to the attractive interactions between the sulfur and oxygen atoms located in the same subsystem. Thus, they show the presence of the intramolecular chalcogen bond in all the studied systems. An interesting situation was found in the case of AZM-TT, where there is also an orange arrow pointing to the green region between the sulfur and nitrogen atoms from the other subsystem. So, in this case, the same sulfur atom (S1) is involved in two chalcogen bonds simultaneously (one intra- and another intermolecular chalcogen bond). The distance between corresponding S and N atoms is 3.38 Å, which is slightly smaller than the sum of their vdW radii (3.55 Å). Furthermore, NCI analysis was performed for geometries extracted from Car–Parrinello Molecular Dynamics that corresponded to (for AZM, AZM-T and AZM-TT): (i) the smallest S1•••O2A distance, (ii) the smallest S1•••O2B distance and (iii) equal S1•••O2A and S1•••O2B distances (see Figure 6). For AZM-TTL and AZM-TTLH as a touchstone of presence of non-covalent interactions, only this geometry was taken for which the S1•••O2A and S1•••O2B distances were closest to the averaged values presented in Table S1. It is evident that in every system from AZM to AZM-TT, two intramolecular chalcogen bonds (S1•••O1 and S1•••O2A or S1•••O2B) were present for the majority of the simulation time—the indication of their presence are green to blue reduced density gradient (RDG) isosurfaces between sulfur and oxygen atoms of the AZM molecule (the intensity is directly correlated with the interaction strength—the more blueish the color, then the more attractive the interaction is). An analysis in the framework of Natural Bond Orbitals (NBO) was also employed and confirmed the existence of S1•••O1 chalcogen bond—associated data and visualization are presented in Supplementary Material (Table S3 and Figure S7). Moreover, the role of the S1 and O1 atoms in providing the anchor points for the presence of NCIs is also visible in the molecular electrostatic potential (MEP) distribution, which is discussed in detail in the Supplementary Material (see Figure S8, Table S4 and associated supporting text).

### 2.4. Atoms in Molecules Analysis of Intra- and Intermolecular Non-Covalent Bonds

AIM is one of the topological tools that enables one to determine whether non-covalent interactions are present in the examined systems by revealing the so-called bond critical points (BCPs) along the path that links two atoms. One may observe that all other CPs than BCPs (ring critical points (RCP) or cage critical points (CCP)) were removed from Figure S5. In the case of S1•••O1 interactions, the BCPs locations were revealed, and various physical quantities corresponding to these points were acquired. The hydrogen bond energy estimation was provided on the basis of Espinosa and Vener models [62,63]. The values of the aforementioned physical quantities for gas phase and for the frame from CPMD selected so that the S1•••O1 distance reached the lowest value are gathered in Table 1. It is valuable to note two things: (i) from time to time, AIM could not find any BCP along the path between S1•••O1 (cases of the geometries corresponding to the largest S1•••O1 distances taken from the Car–Parrinello MD course); (ii) AIM also struggled to find any BCPs between S1 and O2A or O2B (to investigate the possibility of chalcogen bond formation in these cases, the NCI analysis was employed; for more details, see the previous subsection). However, despite the findings mentioned earlier, AIM provides a solid evidence that the intramolecular chalcogen bonds in the studied compounds exist and have a significant impact on the conformation of AZM and its complexes.

Moreover, it can be observed that the increasing number of amino acids of the binding site has pronounced the S1•••O1 non-covalent interaction. This behavior can account for numerous attractive interactions between sulfur and the binding site, which results in more anisotropic charge distribution on the sulfur atom and, as a consequence, a larger area of less negative (or even positive) electrostatic potential. The insight into the molecular electrostatic potential (MEP), as well as the properties of the BCPs not included in Table 1, are gathered and discussed in the Supplementary Material (see Tables S4 and S5, Figure S8).

**Figure 7:**
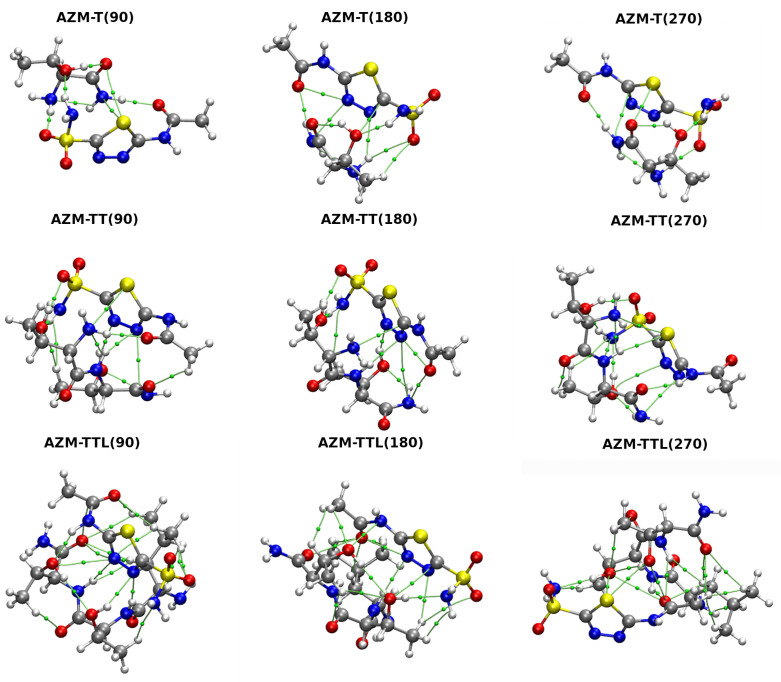
Atoms in Molecules (AIM) molecular graphs for AZM complexes subjected to the S1-C2-N4-C1 torsional angle rotation. Numbers in parentheses denote the torsional angle values in [${}^{\circ }$]. Only BCPs of non-covalent interactions are presented, and they are marked as small green spheres. The simulations were performed at the M06/aug-cc-pVDZ level of theory.

The AIM theory was also employed to investigate properties at BCPs of Car–Parrinello Molecular Dynamics snapshots (see Table S6) and for different values of the S1-C2-N4-C1 torsional angle (see Table S7, Figure 7). Only the data of non-covalent interactions with estimated energy exceeding 2 kcal·mol^−1^ (according to Espinosa equation [62]) were gathered and discussed. Starting with data obtained from analysis of structures taken from the relaxed scan, one can observe that for the AZM-T complex, for certain arrangements of atoms (S1-C2-N4-C1 angle equal to 90, 180, 270 degrees), the sum of energies of the presented non-covalent interactions is far greater than that for the optimized structure. This model shows the importance of an intramolecular, S1•••O1, chalcogen bond presence in the studied systems—this bond not only has a profound impact on the intrinsic structure of AZM alone, but it also affects the AZM interactions with the binding site. A similar tendency was observed for the AZM-TT system, where the energy of interactions for different torsional angle values was very close to the optimized AZM-TT-opt system. In addition, in this case, the chalcogen bond provides a crucial stabilization and has a decisive role in the AZM conformation. The optimal atoms arrangement of AZM-TTL provides already a stronger stabilization in terms of intermolecular non-covalent interactions. Therefore, the S1•••O1 chalcogen bond impact is less pronounced compared to AZM-T and AZM-TT systems.

The analysis of the structures taken from the CPMD run allowed for a qualitative and quantitative influence of intermolecular non-covalent interactions on the strength of the S1•••O1 intramolecular chalcogen bond. It can be noted that for the AZM-T system, the interaction energy of the S1•••O1 chalcogen bond grows almost three times, when the shortest S1•••O1 distance is compared to the optimized structure (both are identified as bonds by the AIM theory; an opposite situation was noted for the largest S1•••O1 interatomic distance). Nonetheless, for this system, an optimized geometry provides a better stabilization due to the presence of numerous weak non-covalent interactions (less than 2 kcal/mol). In the AZM-T(s) complex, the AZM molecule interacts with the binding site through three closed-shell types of interactions, whereas as much as six weaker contacts were discovered when the AIM was applied to the AZM-T optimized structure (see Figure S9 in Supplementary Material). In the AZM-T(l) complex, the O1 and S1 atoms act as acceptors of the hydrogen bond. Moreover, the S1 atom is also involved in the weak interaction with the nitrogen atom from the amino group—due to the presence of these interactions, the S1•••O1 chalcogen bond is not observed.

### 2.5. Partitioning of the Interaction Energy

In order to properly estimate the energies of interactions between AZM and certain parts of the binding site of the Carbonic Anhydrase IX receptor mimic, the Symmetry Adapted Perturbation Theory (SAPT) method was employed (see Table 2).

In the case of the optimized AZM-T structure, it can be observed that the most pronounced attractive contribution stems from the dispersion energy. The same observation can be made for other systems with experimental structures. However, for the AZM-TT, the electrostatic term magnitude in relation to the dispersion grows. It can be simply explained by the polarity of the additional amino acid, threonine, which contains the hydroxyl group in its side chain that can act as a donor or as an acceptor of the hydrogen bond (which, in turn, is mainly electrostatic in nature). In the AZM-TTL complex, the significant growth of the stabilization energy occurs, and this happens due to the electrostatic as well as dispersion component. One may be interested in why the electrostatic contribution grew so much in comparison to the AZM-TT (X-ray data), because AZM-TTL differs from the former only by the hydrophobic amino acid, leucine. However, it should be noted that this peculiarity is observed only in the crystal, and it is probably caused by the specific, non-physical from the view of our simplified system, arrangement of the binding site atoms around the AZM molecule. The outcome of the SAPT calculation of the optimized complexes provides a more intuitive picture of the interaction energy, where the electrostatic contributions of the AZM-TT and AZM-TTL are almost equal in magnitude. Moreover, in the optimized structures, a different trend emerges—for all of the complexes, the main contribution to the stabilization energy is derived from the electrostatic component, not from the dispersion. An explanation of this phenomenon is related to the system complexity: the number of hydrogen bonds formed between the host and the AZM molecule grew significantly in the optimized structures when comparing to the X-ray data.

As it was pointed out earlier, the number of atoms of AZM-TTLH complex prohibited us from employing the SAPT2 level calculations. It can be noted that the overall SAPT0 energy is higher for the AZM-TTLH than for the AZM-TTL when the X-ray structures are taken into consideration. However, the previously mentioned arguments, valid for the AZM-TTL complex, can be put forth. The SAPT0 calculations performed for the optimized structure of the AZM-TTLH give lower total interaction energy and provide evidence that the main attractive energy contribution is the Coulombic term (which is in agreement with other results for smaller systems, which are obtained at a more thorough SAPT2 level of theory). Indeed, in the optimized complexes, the first-order approximation using only the electrostatic and exchange terms does not recover the interaction strength, and the inclusion of induction and dispersion is important for the final outcome. Additional insight into the interaction energy partitioning according to the Morokuma–Ziegler EDA approach [59] at the DFT level of theory is provided in the Supplementary Material—see Table S8 and associated discussion.

## 3. Materials and Methods

### 3.1. First-Principle Molecular Dynamics and Metadynamics Methods

#### 3.1.1. Car–Parrinello Molecular Dynamics (CPMD)

The Car–Parrinello Molecular Dynamics (CPMD) [47] was employed for the gas phase models of AZM molecule and its complexes with amino acids present in the binding pocket of Carbonic Anhydrase IX receptor mimic (see Figure 2). The initial structures of the acetazolamine (N-(5-(aminosulfonyl)-1,3,4-thiadiazol-2-yl)-acetamide—AZM) and amino acids forming the active site of the binding pocket (see Figure 2) were extracted from the complex deposited in the Protein Data Bank (PDB) [64] with entry: 3DC3 [41]. Then, the constructed models of the AZM molecule and its complexes were optimized at the M06/aug-cc-pVDZ level of theory [65,66], and they served for the time-evolution methods as well as static models development. The M06 functional was applied because of its versatility and reliability for applications in thermochemistry and the characterization of non-covalent bonding [65]. In the next step, the initial geometries were taken for the CPMD runs, but they underwent initial structural relaxation (energy minimization) to avoid sudden artificial heating of the nuclear and electronic degrees of freedom. Such an artifact could result when a structure far from equilibrium would be directly used for the CPMD production run. Thus, structure relaxation was carried out using the Perdew–Burke–Ernzerhof functional with Grimme’s dispersion corrections denoted as PBE-D2 [67,68] and with the Troullier–Martins norm-conserving pseudopotentials [69]. An initial estimate of the Hessian matrix for the energy minimization was performed using the Schlegel method [70]. The simulations were carried out in cubic boxes with a = 20 Å. The Hockney method was employed to achieve the gas phase conditions and the Γ-point approximation was used to reduce the computational cost and memory usage. A kinetic energy cutoff of 100 Ry was applied for the plane-wave basis set. Next, the molecular dynamics simulations were performed with the NVT ensemble. The time step was set to 3 a.u. (0.0725 fs), and a fictitious electron mass parameter (EMASS) of 400 a.u. was applied to reproduce the orbital dynamics. The computations were performed at 297 K, and the Nosé–Hoover thermostat was used to maintain the specified conditions [71]. The initial part of the MD run was taken as an equilibration phase (10,000 steps) and was not considered during the data analysis. The trajectories of 108 ps (production run) were collected and further used to investigate metric parameters of chalcogen and hydrogen bonds in the AZM molecule and its complexes.

In addition, separate CPMD runs were carried out for the AZM molecule and selected smaller complexes with amino acids to show the electronic structure evolution as a function of time. A localization procedure for the molecular orbitals was invoked, yielding the optimally localized Wannier functions [72] every 1000 steps of the run. The setup for these CPMD simulations was exactly the same as described above. The CPMD simulations were performed using the 4.3 version of the CPMD program [73].

#### 3.1.2. Path Integral Molecular Dynamics (PIMD)

Path Integral Molecular Dynamics (PIMD) [48,60] was carried out for the AZM molecule and its complexes with amino acids forming the binding pocket (the model preparation was described in the previous section). The PIMD simulations were performed using a setup similar to the CPMD runs. The idea of the PIMD application is associated with the qualitative/quantitative description of the quantum effects inclusion and their influence on the chalcogen and intermolecular hydrogen bonding in the studied systems. The simulations were carried out at T = 297 K controlled by Nosé–Hoover thermostat [71]. Eight Trotter replicas (P = 8) were applied for imaginary time path integration. The staging representation of the path integral propagator was used [74]. The investigated systems were placed into cubic boxes with a = 16 Å for the AZM molecule and 20 Å for its complexes. The initial 10,000 steps of the simulation time served as an equilibration phase, while 34 ps were taken as the production run and further analyzed. The PIMD computations were carried out using the 4.3 version of the CPMD program [73].

#### 3.1.3. Metadynamics (METD)

In order to reproduce the free energy (F) profile of the AZM chalcogen bond and intermolecular hydrogen bond formed between the AZM molecule and amino acids deriving from the binding pocked, the metadynamics (METD) method was applied [50,75]. For the electronic structure description, the CPMD setup as described above was used. The 200,000 CPMD steps were employed, and a placement of a new Gaussian-like hill in the potential energy of the studied complexes (in the Gaussian tube formalism, the hill height was 0.001 a.u.) took place between every 500 and 1000 CPMD steps (dependent on the movement of the collective variables, CVs). The initial velocities of the collective variables were initialized to provide 297 K temperature, and a velocity rescaling algorithm was used to maintain these conditions for the CVs. Several choices of the CVs were tested, and the most successful have been: S1-C2-N4-C1 and S1-C3-S2-O2B dihedral angles (see Figure 2 for atoms numbering scheme), which are responsible for the intramolecular chalcogen bond and orientation of the interaction donors and acceptors within the ligand with respect to the environment. The metadynamics simulations were performed with the CPMD version 4.3 program [73].

#### 3.1.4. Post-Processing of the Results Based on Time-Evolution Methods

The analysis of the CPMD, PIMD and METD results was performed with the usage of 5.2 and 1.9.3 versions of the Gnuplot [76] and Visual Molecular Dynamics (VMD) [77] programs, respectively. Spectroscopic signatures were obtained by Fourier transform of the autocorrelation function of atomic velocity for all atoms spectra, and for chosen functional groups. The computed spectra were decomposed using home-made scripts written in the Fortran programming language [78]. Additionally, the post-processing of the PIMD and METD results was carried out based on home-made scripts written in Fortran as well. In order to investigate the non-covalent interactions dynamical features, snapshots from the CPMD run were analyzed. The conformations corresponding to S1•••O1, S1•••O2A and S1•••O2B distances (the shortest, average and the longest) were extracted and subsequently analyzed with static approaches based on NCI and AIM frameworks. The wavefunctions for the non-equilibrium structures were obtained as a result of single point calculations at the M06/aug-cc-pVDZ level of theory.

### 3.2. Density Functional Theory (DFT) and Post-Hartree–Fock Methods

The energy minimization of the investigated AZM molecule and its complexes with amino acids was performed on the basis of Density Functional Theory (DFT) [79,80]. The meta-GGA and M06 functional [65] with Dunning’s type basis set denoted as aug-cc-pVDZ [66] were employed because of their good performance at modeling of non-covalent interactions with the DFT framework [81]. Harmonic frequencies were computed to confirm that the obtained structures correspond to the minimum on the Potential Energy Surface (PES). In order to estimate the chalcogen bond energy, the relaxed scan method (with 10${}^{\circ }$ increment) was applied, and the S1-C2-N4-C1 torsion angle was taken into account during the simulations. We have tested diverse levels of theory (based on DFT and post-Hartree–Fock) to reproduce the potential energy barriers. The MP2/aug-cc-pVDZ relaxed scan geometries were extracted and served as reference structures for the CCSD/aug-cc-pVDZ and CCSD(T)/aug-cc-pVTZ single point calculations. Next, the wavefunctions for the Atoms in Molecules (AIM) study [53] were obtained for equilibrium and non-equilibrium structures by employing the same (M06/aug-cc-pVDZ) computational setup. The non-equilibrium structures, either derived from rotational profiles of the selected dihedral angles or sampled from the CPMD trajectory, served for exploration of the phase space and finding the diversity of the interaction patterns. It is necessary to point out that the AIM formalism should be used at the equilibrium geometry, so that the standard nomenclature related to Bond and Ring Critical Points (BCPs and RCPs) could be used. In the present study, non-equilibrium structures are studied, and the electronic and topological properties cannot be strictly related to the terminology used in the classical AIM formulation. However, we label the located critical points as BCPs and RCPs following the convention frequently used in numerous publications of non-equilibrium structures where AIM theory was employed to facilitate the results discussion [82,83]. This part of the computations was carried out with the Gaussian 16 Rev. C.01 suite of programs [84]. In the next step, the electronic structure analyses were performed on the basis of the Non-Covalent Interaction (NCI) index [56,85] and Natural Bond Orbital (NBO) theory [54,55,86]. The NBO theory was engaged to reveal the parameters regarding orbital interaction energies. The NCI index was estimated using the MultiWFN program [87,88]. The NBO analysis was carried out with assistance of the NBO 3.1 software [89]. The decomposition of the interaction energies was achieved using the M06 [65] and PBE0 [90] functionals with the TZ2P basis set [91] through the ADF–EDA protocol according to the Morokuma–Ziegler scheme embedded in the ADF software [92,93,94]. The Molecular Electrostatic Potential (MEP) was computed at the M06/aug-cc-pVDZ level of theory for the investigated systems to find the electrostatic potential extrema on the 0.001 a.u. electronic isodensity contour using the MultiWFN program [87,88]. The MEP visualization was performed based on the VMD software [77]. The Atoms in Molecules (AIM) theory [53] was employed for electronic structure and topology study of the AZM molecule and its complexes with amino acids. This theoretical approach allowed specifically gaining an insight into the electron density distribution changes (as the peptide chain growths) and the possible intra- and inter- non-covalent interactions [95,96]. On the basis of the AIM theory, the locations of the Bond Critical Points (BCPs) were revealed in the investigated structures. The electronic structure features at these points were determined using electron density ρ and its Laplacian $\nabla^{2}$ρ. The Lagrangian kinetic energy density ($G_{\mathrm{CP}}$) as well as potential energy density were computed ($V_{\mathrm{CP}}$) at the critical points in order to enable the determination of bonding properties [95,96]. The energy of the non-covalent interactions was approximated using the Espinosa and Vener formulas [62,63]: $E_{int}$ = $-\frac{1}{2}$$V_{\mathrm{CP}}$(r) and $E_{int}$ = 0.492 $G_{\mathrm{CP}}$(r), respectively, where $V_{\mathrm{CP}}$(r) and $G_{\mathrm{CP}}$(r) are potential energy density and Lagrangian kinetic energy density evaluated at the relevant BCP. The values of the Lagrangian kinetic energy density are not reported in the relevant table. The AIM analysis was carried out with assistance of the MultiWFN program [87,88].

### 3.3. Symmetry-Adapted Perturbation Theory (SAPT)

Interaction energy between the AZM molecule and the amino acids forming the binding pocket was evaluated within the SAPT scheme [57]. The structures of the complexes: AZM-T, AZM-TT, AZM-TTL and AZM-TTLH (which can be considered as supramolecular dimers) were taken into consideration. The SAPT was applied for two kinds of dimers:

1. Extracted from the Carbonic Anhydrase IX mimic–AZM complex (PDB deposit 3DC3) and
2. Optimized at the M06/aug-cc-pVDZ level of theory [65,66].

The SAPT0/aug-cc-pVDZ and SAPT2/aug-cc-pVDZ levels of theory were chosen for the energy decomposition between the dimers. Density fitting (RI and JKI) with aug-cc-pVDZ as auxiliary basis sets were performed in order to approximate four-index integrals. The studied dimers were separated into two monomers (the ligand as the first monomer and the growing amino acid chain as the second monomer) to fulfill the conditions required to apply the counterpoise correction method by Boys and Bernardi [97] needed to eliminate the basis set superposition error (BSSE). The calculations were performed using the Psi4 1.3.2 program [98].

## 4. Conclusions

A comprehensive study of the acetazolamide (AZM) molecule and its interactions with the Carbonic Anhydrase IX mimic binding site models was carried out using state-of-the-art theoretical methods. The combination of dynamical and static approaches was devoted to the detailed description of the new class of non-covalent chalcogen bonds. Due to the nature of this type of interactions, it was challenging to employ Car–Parrinello molecular dynamics, Path Integral MD and metadynamics schemes. The most important findings are related to the origins of the conformational preferences of the ligand and the nature of its interactions with the amino acid residues. The S1•••O1 chalcogen bond in the AZM molecule is an important factor stabilizing the preferred conformation together with the N3-H3A•••N2 and N3-H3B•••N2 contacts. The isolated AZM is still conformationally flexible—the presence of intermolecular contacts with the amino acid residues is crucial in restricting ligand mobility in the binding site, as revealed by the CPMD, metadynamics and static approaches. Nuclear quantum effects from both protons and heavier nuclei, modeled with the Path Integral method, were found to affect the conformational landscape of the studied complexes. The ligand–amino acid interactions, analyzed within the SAPT framework, are dominated by an electrostatic term corresponding to the growing number of hydrogen bonds. Recent state-of-the-art research on the various manifestations of the non-covalent interactions significance for the complex systems has showed the need for deeper studies based on First-Principle Molecular Dynamics.

## Figures and Tables

**Table 1:** AIM-derived properties at BCPs along S1•••O1 path taken from the structures optimized at the M06/aug-cc-pVDZ level of theory and from the CPMD frame for which the S1•••O1 distance was the shortest. E1 and E2 are bond energies based on Espinosa and Vener models, respectively. The dimensions of given quantities are as follows: electron density, $\rho_{BCP}$, is given in $e\cdot a_{0}^{-3}$ atomic units and the Laplacian of electron density, $\nabla^{2}\rho_{BCP}$, is in $e\cdot a_{0}^{-5}$ units. V_CP_ stands for BCP potential energy density and G_CP_ denotes the Lagrangian of kinetic energy density at the BCP.

Complex	Origin	ρ	$\nabla^{2}$ ρ	V_CP_	G_CP_	E1	E2
AZM	DFT	0.020	0.066	−0.014	0.015	4.478	4.132
	CPMD	0.045	0.152	−0.040	0.039	12.490	10.486
AZM-T	DFT	0.020	0.065	−0.014	0.015	4.426	4.086
	CPMD	0.049	0.163	−0.045	0.043	14.018	11.490
AZM-TT	DFT	0.021	0.067	−0.015	0.016	4.568	4.210
	CPMD	0.053	0.173	−0.049	0.046	15.249	12.370
AZM-TTL	DFT	0.022	0.071	−0.016	0.017	4.940	4.520
	CPMD	0.052	0.164	−0.047	0.044	14.605	11.773
AZM-TTLH	DFT	0.022	0.072	−0.016	0.017	4.953	4.533
	CPMD	0.054	0.170	−0.049	0.046	15.445	12.341

**Table 2:** Interaction energy partitioning (in kcal/mol) obtained at the SAPT0/aug-cc-pVDZ and SAPT2/aug-cc-pVDZ levels of theory. The exception was the AZM-TTLH complex, which was too large to compute its energies at the SAPT2 level of theory.

Complex	Electrostatics	Exchange	Induction	Dispersion	SAPT0	SAPT2
	Experimental structure					
AZM-T	−5.846	13.985	−2.603	−7.157	−2.979	−1.621
AZM-TT	−9.853	19.973	−4.291	−11.171	−6.599	−5.342
AZM-TTL	−18.854	30.558	−8.371	−20.057	−19.657	−16.723
AZM-TTLH, SAPT0	−12.516	29.249	−8.447	−22.552	−14.266	–
	Optimized structure					
AZM-T	−22.738	26.825	−8.096	−13.031	−20.543	−17.040
AZM-TT	−40.280	51.954	−15.134	−26.006	−36.737	−29.465
AZM-TTL	−40.443	51.785	−15.710	−30.315	−41.111	−34.684
AZM-TTLH, SAPT0	−68.386	69.536	−26.657	−40.273	−65.780	–

## Data Availability

The data presented in the current study are available in the article and in the associated Supplementary Material.

## Supplementary Materials

The file containing supplementary Tables and Figures is available online at https://www.mdpi.com/article/10.3390/ijms232213701/s1. Reference [99] is cited in Supplementary Materials.

## Abbreviations

The following abbreviations are used in this manuscript:

AZM	Acetazolamide
FPMD	First-Principle Molecular Dynamics
METD	Metadynamics
DFT	Density Functional Theory
NCI	Non-Covalent Interaction
NBO	Natural Bond Orbital
AIM	Atoms In Molecules
SAPT	Symmetry-Adapted Perturbation Theory
CPMD	Car–Parrinello Molecular Dynamics
PIMD	Path Integral Molecular Dynamics
HB	Hydrogen bond
EDA	Energy Decomposition Analysis
PDB	Protein Data Bank
CV	Collective Variable
PES	Potential Energy Surface
BCP	Bond Critical Point
RCP	Ring Critical Point
MEP	Molecular Electrostatic Potential
BSSE	Basis Set Superposition Error
NQE	Nuclear Quantum Effects
