# Supplementary material for: Chalcogen Bond as a Factor Stabilizing Ligand Conformation in the Binding Pocket of Carbonic Anhydrase IX Receptor Mimic
Source: Int J Mol Sci. 2022 Nov 8;23(22):13701. doi: 10.3390/ijms232213701 (PMC9691181; doi:10.3390/ijms232213701)
Supplement: Supplementary file 1 [file ijms-23-13701-s001.zip › ijms-1944739-supplementary.pdf]

SUPPLEMENTARY MATERIAL

Chalcogen bond as a factor stabilizing ligand conformation in the binding pocket of Carbonic Anhydrase IX receptor mimic

Kamil Wojtkowiak[1*], Mariusz Michalczyk[2*], Wiktor Zierkiewicz[2], Aneta Jezierska[1], Jarosław J. Panek[1]

[1] University of Wrocław, Faculty of Chemistry, ul. F. Joliot-Curie 14, 50-383 Wrocław, Poland

[2] Wrocław University of Science and Technology, Faculty of Chemistry, Wybrzeże Wyspiańskiego 27, 50-370 Wrocław, Poland

Correspondence should be addressed to:

Kamil Wojtkowiak; e-mail: kamil.wojtkowiak@chem.uni.wroc.pl

Mariusz Michalczyk; e-mail: mariusz.michalczyk@pwr.edu.pl

Table of contents:

I. **Table S1.** Comparison of metric parameters related to the chalcogen bond taken from the experimental data (PDB code: 3DC3 [1]), the averaged values taken from the CPMD runs and optimized at the M06/aug-cc-pVDZ level of theory. The interatomic distances are given in [Å] while the angles are in [°].

II. **Figure S1.** Time-evolution of interatomic distances of atoms involved in the S1•••O1 intramolecular chalcogen bond formation as a result of the CPMD simulations of the AZM molecule and its complexes with amino acids. The averaged values of the chalcogen bond are given.

III. **Figure S2.** Time-evolution of interatomic distances of atoms involved in diverse non-covalent interactions present in the AZM-T, AZM-TT, AZM-TTL and AZM-TTLH complexes (from top to bottom).

IV. Figure S3. Time-evolution of interatomic distances of atoms involved in the S1•••O2A and S1•••O2B intramolecular chalcogen bonds formation as a result of the CPMD simulations of the AZM molecule and its complexes with amino acids.

V. Figure S4. Power spectra of atomic velocity of all atoms of the AZM molecule (left panel) and the contribution of the C=O group from the AZM molecule and its complexes with amino acid (right panel) as a result of the CPMD simulations.

VI. Figure S5. Potential energy profiles obtained as a result of the S1-C2-N4-C1 dihedral angle variation of the AZM molecule. The simulations were performed at various levels of theory (for details see **Table S2**).

VII. Table S2. Relative energy barrier in [kcal/mol] as a result of the S1-C2-N4-C1 dihedral angle rotation of the AZM molecule. The energy estimation was carried out on the basis of the relaxed scan results.

VIII. Table S3. Interaction energy (E2) between lone electron pairs on the O1 oxygen atom and antibonding σ*(C3–S1) orbital (kcal/mol) in the studied complexes. The calculations were performed at the M06/aug-cc-pVDZ level of theory.

IX. Figure S6. NCI diagrams of the investigated systems. Colors represent various type of interaction: green – interaction of average strength, brown – very weak interactions, red – repulsion. Black arrows point to the green regions corresponding to the intramolecular chalcogen bond (between oxygen and sulfur atoms), while orange ones indicate at intermolecular chalcogen bond (between oxygen and nitrogen atoms).

X. Figure S7. The isosurface (on the 0.03 isovalue) of the interacting orbitals LP1(O1) or LP2(O1) and $\sigma*$(C3-S1) in the AZM molecule. The LP(O1) orbitals are violet-colored, while $\sigma*$(C3-S1) is orange.

XI. Figure S8. Molecular Electrostatic Potential (MEP) isosurfaces on the 0.001 a.u. isodensity surface computed at the M06/aug-cc-pVDZ level of theory for the AZM molecule and its complexes with amino acids. Scale: from -0.01 (blue) to 0.01 a.u. (red). The two extremes are marked with black dots.

XII. Table S4. Values of selected extremes on 0.001 a.u. isodensity MEP surface calculated at the M06/aug-cc-pVDZ level of theory for the studied AZM molecule and its complexes with amino acids.

XIII. Table S5. AIM-derived properties at BCPs of intermolecular non-covalent interactions of equilibrium structures. $E1_{HB}$ and $E2_{HB}$ are intermolecular hydrogen bond energies [kcal/mol] based on Espinosa and Vener equation. Electron density, ρ_{BCP}, is given in $e{*}a_0^{-3}$

and its Laplacian, $\nabla^2\rho_{BCP}$, in $e^*a_0^{-5}$. V_{CP} and G_{CP} denote potential and kinetic energy density at the BCPs respectively.

XIV. Table S6. AIM-derived properties at BCPs of the investigated systems (excluding S1•••O1 chalcogen bond) taken from the CPMD runs. The $E1_{HB}$ and $E2_{HB}$ are intermolecular hydrogen bond energies [kcal/mol] based on Espinosa and Vener equation. Electron density, ρ_{BCP}, is given in $e^*a_0^{-3}$ and its Laplacian, $\nabla^2\rho_{BCP}$, in $e^*a_0^{-5}$. V_{CP} and G_{CP} denote the potential and kinetic energy density at the BCPs respectively. AZM-T(s) and AZM-T(l) denote conformations where the S1•••O1 chalcogen bond was the shortest and the longest, respectively.

XV. Table S7. AIM-derived properties at BCPs of the investigated complexes (excluding the S1•••O1 chalcogen bond). The number next to the system designation describes the rotation along the S1-C2-N4-C1 torsional angle. Relaxed scan was performed at the M06/aug-cc-pVDZ level of theory with S1-C2-N4-C1 torsional angle rotation as a variable. E1 and E2 are hydrogen bond energies computed based on Espinosa and Vener equation. The dimensions of given quantities are as follows: ρ_{BCP} is given in $e^*a_0^{-3}$, its Laplacian, $\nabla^2\rho_{BCP}$, in $e^*a_0^{-5}$, whereas V_{CP} and G_{CP} denote the potential and kinetic energy density at the BCPs respectively.

XVI. Figure S9. Atoms in Molecules (AIM) molecular graphs of the AZM molecule and its complexes with amino acids. The simulations were performed at the M06/aug-cc-pVDZ level of theory. Only BCPs of non-covalent interactions are presented and marked as small green spheres along bond paths.

XVII. Table S8. Decomposition of the interaction energy of complexes into Pauli repulsion (E_{pauli}), electrostatic (E_{elec}), orbital interaction (E_{oi}) and dispersion (E_{disp}) components. All energies in kcal/mol.

Table S1. Comparison of metric parameters related to the chalcogen bond taken from the experimental data (PDB code: 3DC3 [1]), the averaged values taken from the CPMD runs and optimized at the M06/aug-cc-pVDZ level of theory. The interatomic distances are given in [Å] while the angles are in [°].

Metric parameters	AZM	AZM-T	AZM-TT	AZM-TTL	AZM-TTLH
**Experimental structure (X-ray measurement data)**					
**S1•••O1**	2.972	-	-	-	-
**∠C3-S1•••O1**	165.75	-	-	-	-
**∠S1-C2-N4-C1**	-36.342	-	-	-	-
**S1•••O2A**	3.026	-	-	-	-
**S1•••O2B**	3.910	-	-	-	-
**∠S1-C3-S2-O2A**	7.52	-	-	-	-
**∠S1-C3-S2-O2B**	-116.82	-	-	-	-
**Averaged values from the CPMD runs**					
**S1•••O1**	2.799	2.820	2.714	2.737	2.725
**∠C3-S1•••O1**	155.23	154.80	156.76	156.83	156.43
**∠S1-C2-N4-C1**	-1.59	5.53	3.13	2.37	2.25
**S1•••O2A**	3.510	3.729	3.623	3.226	3.268
**S1•••O2B**	3.605	3.380	3.493	4.072	4.168
**∠S1-C3-S2-O2A**	55.37	85.09	72.26	2.76	-29.65
**∠S1-C3-S2-O2B**	-67.86	-46.02	-54.73	-128.64	-159.98

Table S1 (Continuation). Comparison of metric parameters related to the chalcogen bond taken from the experimental data (PDB code: 3DC3 [1]), the averaged values taken from the CPMD runs and optimized at the M06/aug-cc-pVDZ level of theory. The interatomic distances are given in [Å] while the angles are in [°].

Metric parameters	AZM	AZM-T	AZM-TT	AZM-TTL	AZM-TTLH
**M06/aug-cc-pVDZ level of theory**					
**S1•••O1**	2.751	2.757	2.740	2.705	2.704
**∠C3-S1•••O1**	156.88	156.80	155.68	156.89	157.21
**∠S1-C2-N4-C1**	-0.59	-1.00	4.19	3.02	-3.96
**S1•••O2A**	3.665	3.220	3.861	3.174	3.198
**S1•••O2B**	3.387	4.236	3.164	4.065	4.166
**∠S1-C3-S2-O2A**	80.60	-27.48	95.15	6.87	-15.37
**∠S1-C3-S2-O2B**	-51.70	-158.67	-34.96	-125.62	-144.60

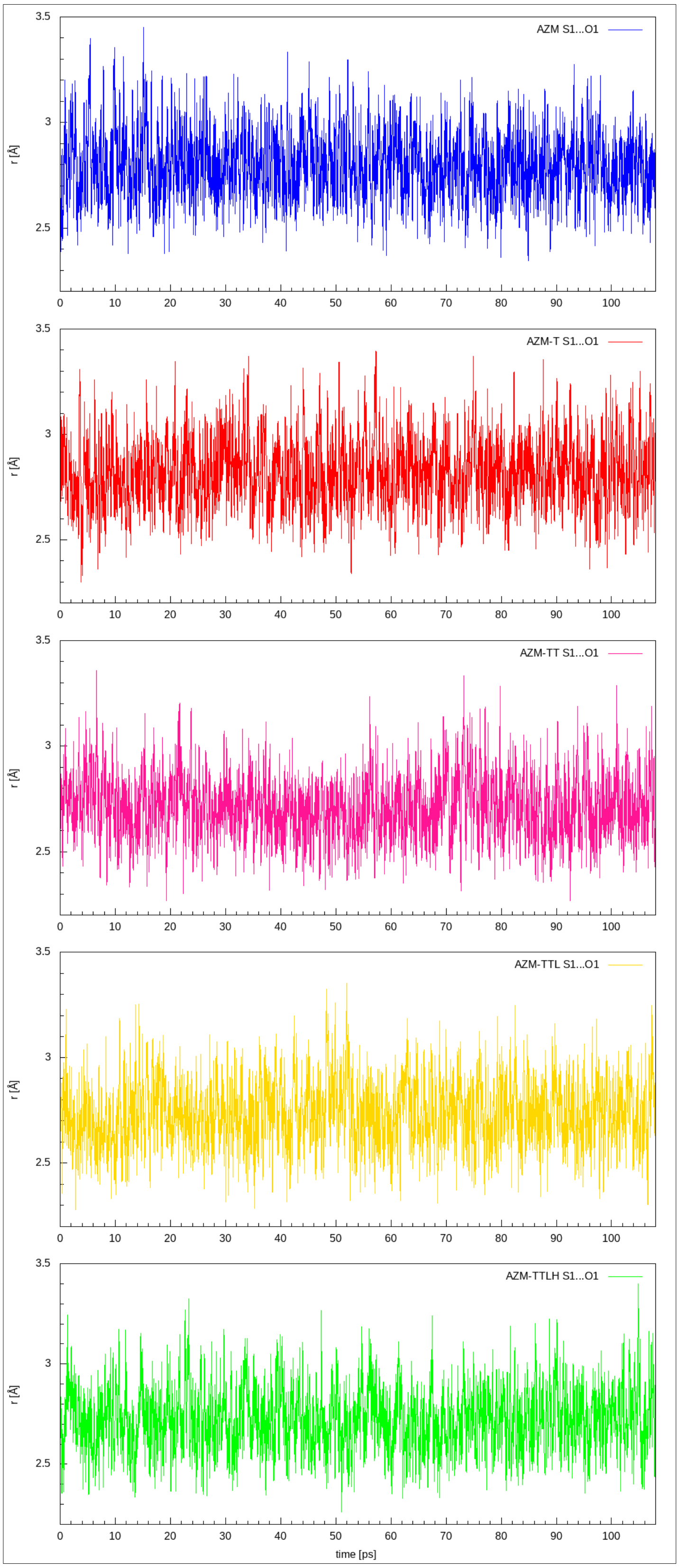

S1•••O1
average 2.799 Å

S1•••O1
average 2.820 Å

S1•••O1
average 2.714 Å

S1•••O1
average 2.737 Å

S1•••O1
average 2.725 Å

Figure S1. Time-evolution of interatomic distances of atoms involved in the S1•••O1 intramolecular chalcogen bond formation as a result of the CPMD simulations of the AZM molecule and its complexes with amino acids. The averaged values of the chalcogen bond are given.

Throughout the simulations the distance between the Lewis acid (S1) and the Lewis base (O1) fluctuated giving an average value of 2.714 Å for the AZM-TT and 2.820 Å for AZM-T (see Table S1 and Figure S1 for graphical presentation of the results). These are the values of the shortest and the longest chalcogen bond present in the studied complexes. The averaged values of the S1•••O1 bond length suggest that the environment does not have a direct impact on the bond parameters. The conclusion is supported by the fact that the bond lengths, valence angles as well as torsional angles were changing in similar manner in each of the examined complexes. The presence of the S1•••O1 chalcogen bond imposes an important constraint on the investigated molecule conformational flexibility. It should be considered as a significant factor stabilizing the structure of AZM.

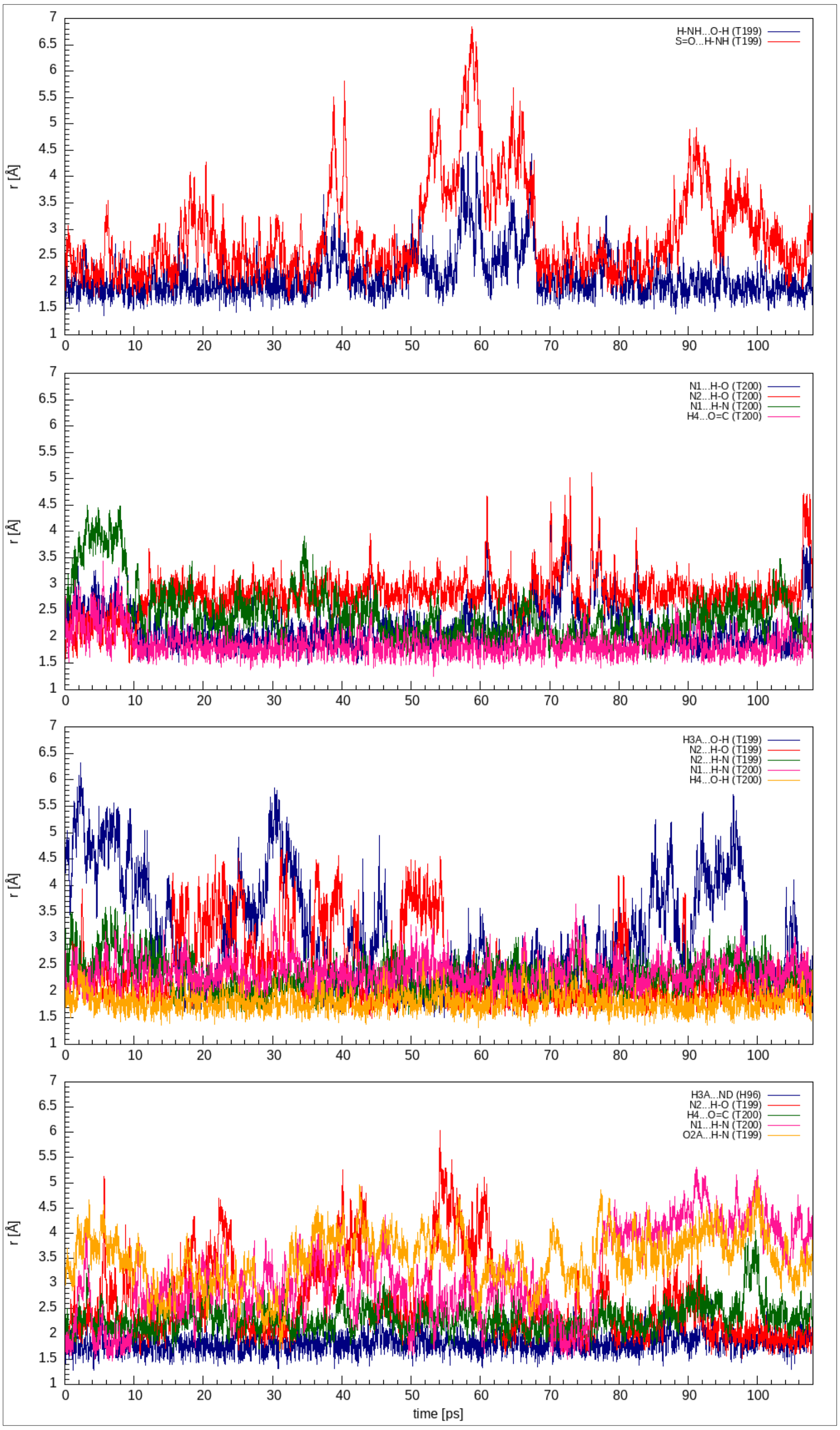

Figure S2. Time-evolution of interatomic distances of atoms involved in diverse non-covalent interactions present in the AZM-T, AZM-TT, AZM-TTL and AZM-TTLH complexes (from top to bottom).

Intermolecular interactions metric parameters were extracted from the CPMD trajectory. The conformation of AZM was stabilized by formation of the hydrogen bonds with the binding site. One may observe that the N1, N2, H3A and H4 atoms are involved in the secondary bonds in each of the investigated complexes. The interactions present throughout the whole CPMD simulation time were: (i) for AZM-TT: the interactions of N1•••H-O (T200) and H4•••O=C (T200); (ii) for AZM-TTL: H4•••O-H (T200), N1•••H-N (T200), N2•••H-N (T199) interactions; (iii) for AZM-TTLH: H4•••O=C (T200) and H3A•••ND (H96).

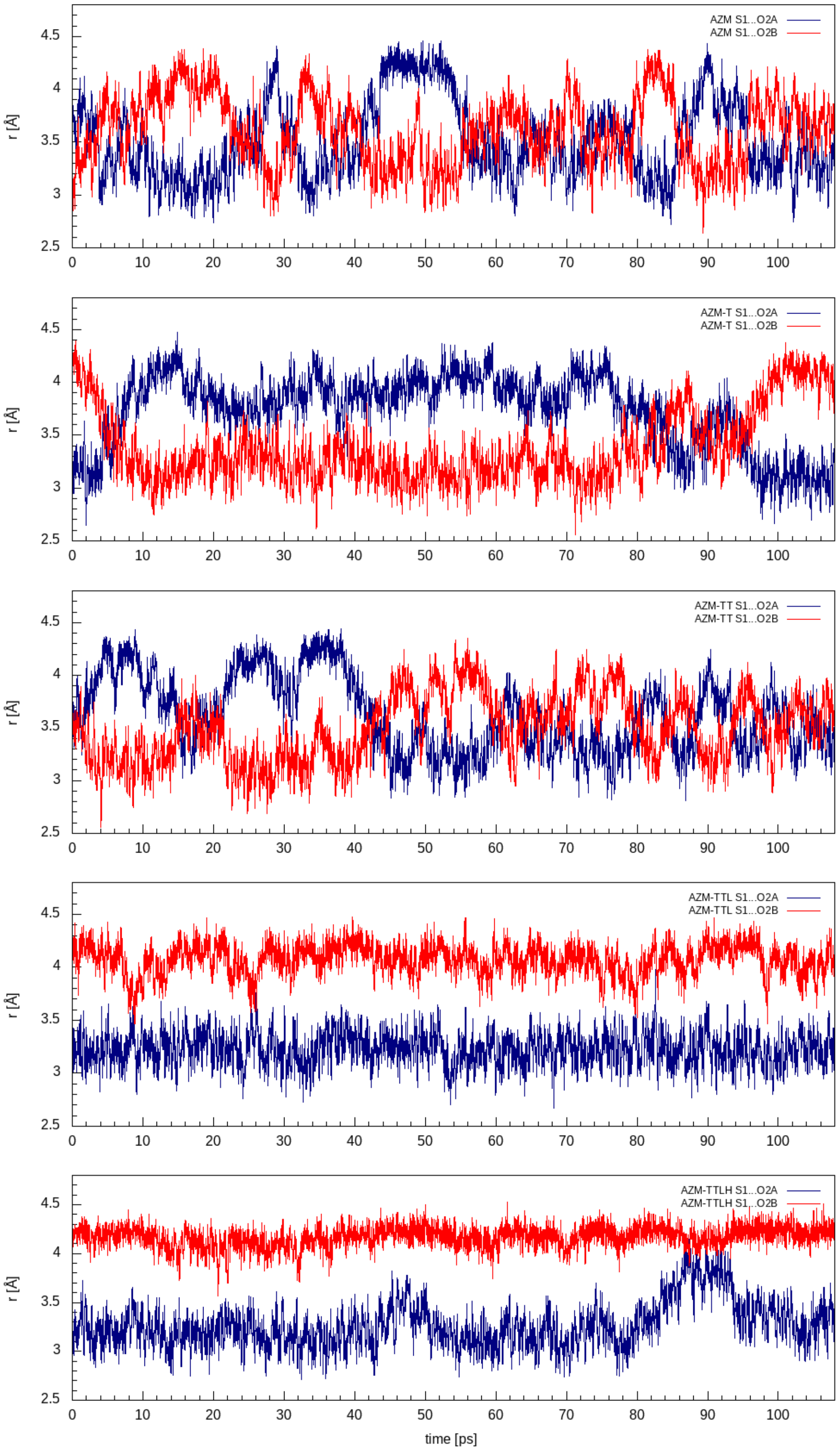

Figure S3. Time-evolution of interatomic distances of atoms involved in the S1•••O2A and S1•••O2B intramolecular chalcogen bonds formation as a result of the CPMD simulations of the AZM molecule and its complexes with amino acids.

Metric parameters between the S1 and O2A and O2B atoms were also measured and gathered in Table S1. As the data have shown, two Lewis bases (O2A and O2B) were interchanging their positions with respect to the acceptor of the electron density (S1 atom). The interchange could not be observed and would be easily overlooked with traditional approaches applied to the secondary bonds investigations - due to the characteristics of static methods, they would either show only statistically averaged quantities for a specific conformation (when the system taken from the crystal would be studied) or they would not consider the impact of the temperature (structures after standard energy minimization). However, the obtained data from the CPMD run could not provide a definite answer, if the S1•••O2A or S1•••O2B chalcogen bonds exist taking into account only the metric parameters. In order to answer the question, a complementary static approach, based on Non-Covalent Interaction (NCI) index, was employed (see Figure 6 of the main text). One can observe the significant conformational flexibility of the S1-C3-S2-O2A dihedral angle (from ca. -90 to 90°) throughout the whole simulation time in the AZM molecule, AZM-T and AZM-TT complexes. The NCI analysis provided an evidence for the reason of this phenomenon and it is twofold: firstly, the presence of the chalcogen bond between S1 and O2A or O2B atoms, and secondly, the H3A•••N2 (or H3B•••N2) intramolecular hydrogen bonds formation. In the AZM-TTL and AZM-TTLH complexes the situation was different due to the numerous non-covalent interactions of the AZM with amino acids of the binding site - for them the S1•••O2A interatomic distance was shorter and no “interchanging” was observed.

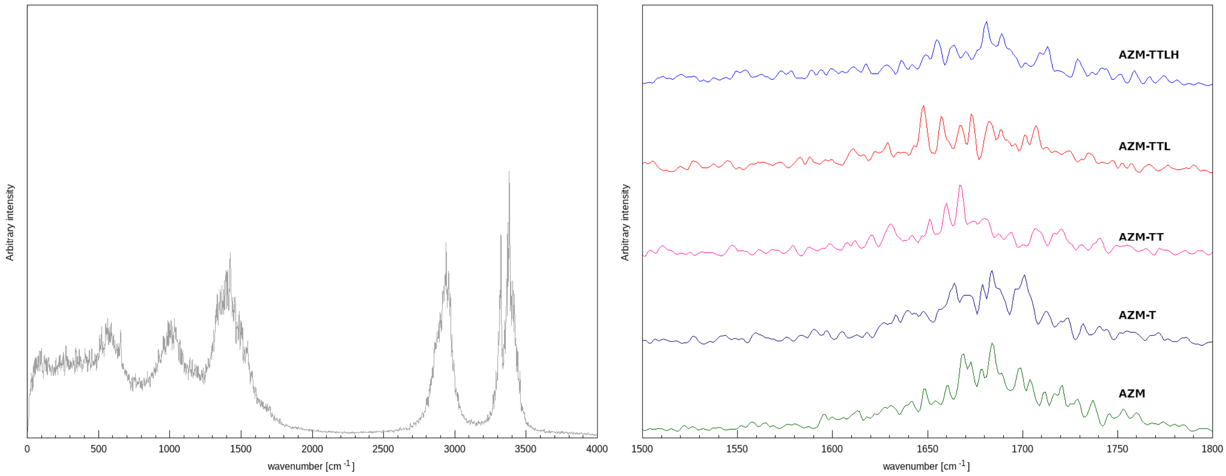

Figure S4. Power spectra of atomic velocity of all atoms of the AZM molecule (left panel) and the contribution of the C=O group from the AZM molecule and its complexes with amino acid (right panel) as a result of the CPMD simulations.

The contribution of the carbonyl group to the power spectra of atomic velocity was derived from CPMD for each of the studied complexes. One should note that the strength of every non-covalent interaction can be associated with the red-shift present on the IR spectrum – the bond between a Lewis base and an atom, to which it is attached (it will elongate and its vibrational frequency will be lowered, when the associated closed-shell interaction of LA•••LB grows in the strength [2]). From the power spectra it can be assumed that the S1•••O1 interaction is the most pronounced for AZM-TTL, AZM-TT and AZM-TTLH complexes, where the centers of the C=O stretching bands are red-shifted for ca. 25-30 cm^{-1}, 20 cm^{-1} and 10 cm^{-1}, respectively, when compared to the AZM (for which the amide I band is present at ca. 1685 cm^{-1}). On the contrary, in the case of AZM-T, the blue-shift of ca. 10 cm^{-1}, with respect to the AZM, can be observed. Thus, conclusions drawn from the power spectra results are not consistent with other methods applied to study the S1•••O1 interaction strength (e.g. AIM, NBO). When static methods are taken into consideration, one would expect that the C=O bands of AZM-TTLH complex will be shifted to the significantly lower wavenumbers than its counterparts from AZM-T or AZM. In the case of larger complexes, other intermolecular contacts between the O1 atom and the neighborhood could easily disrupt the correlation between the red-shift and the strength of the inspected secondary bond.

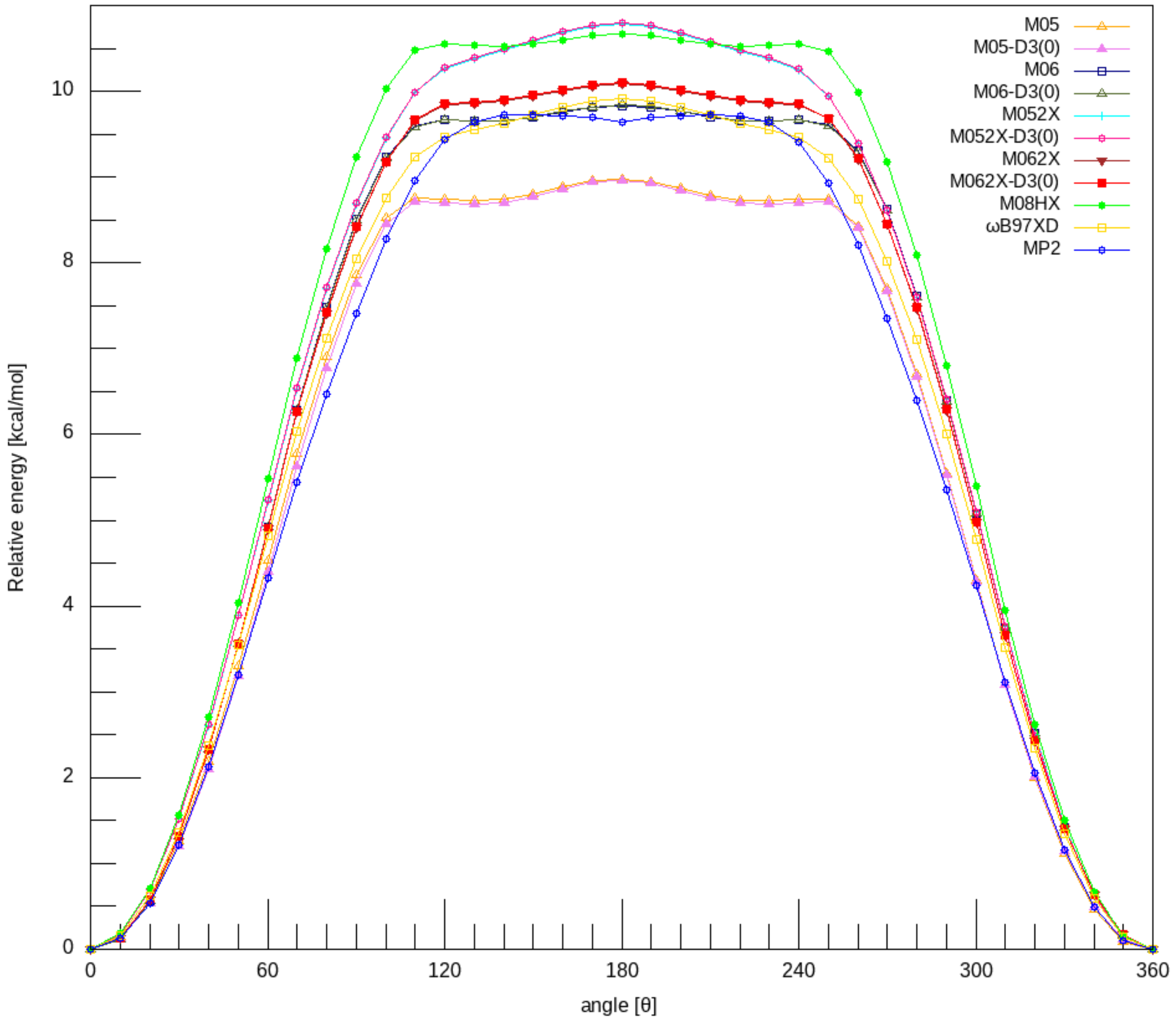

Figure S5. Potential energy profiles obtained as a result of the S1-C2-N4-C1 dihedral angle variation of the AZM molecule. The simulations were performed at various levels of theory (for details see **Table S2**).

In order to estimate the energy of the intramolecular chalcogen bond present in the AZM ligand the relaxed potential energy surface scan has been performed. The torsional angle S1-C2-N4-C1 was varied and the relative potential energy values associated with scan of the dihedral angle are depicted in Figure S5. In addition, the values of the relative energies are presented in Table S2. When scanned coordinate was a torsional angle S1-C2-N4-C1 for almost all cases the least and most stable conformations were associated with the 180 and 0 degrees, respectively. An exception of the observation was the result obtained at the MP2/aug-cc-pVDZ level of theory for which the highest energy was noticed at 140 degrees. Nonetheless, to be consistent with the assumed methodology, the value of the energy at 180

degrees was taken in this case to calculate S1•••O1 bond energy – having that in mind, the corresponding geometry obtained at the MP2/aug-cc-pVDZ level was used for single-point energy calculations using CCSD/aug-cc-pVDZ and CCSD(T)/aug-cc-pVTZ methods. Considering the CCSD(T)/aug-cc-pVTZ//MP2/aug-cc-pVDZ as the reference, the MP2/aug-cc-pVDZ level was the closest for the proper energy estimation. Unfortunately, the scaling of the MP2 method prevented us from performing the energy minimization at this level of theory for larger complexes discussed in this paper. It is important to underline the fact that the functional M06-2X, considered as the best when it comes to study non-covalent interactions, with Grimme D3 dispersion corrections (with zero-damping function) performed very inconsistently in this case (the same problem occurred for M05-2X and M06-2X without dispersion corrections) – one can observe the huge over-binding effect, in comparison with CCSD(T)/aug-cc-pVTZ, when it comes to the estimation of the S1•••O1 chalcogen bond energy. The same effect has been observed for the M08-HX functional. To sum up, encouraged by the results obtained at the M06/aug-cc-pVDZ level of theory and being aware of the complications that can be introduced by the post-SCF dispersion correction schemes in the context of heavily-parametrized Minnesota functionals [1], we decided to continue with M06 as the exchange-correlation functional for geometry optimizations for the remaining complexes.

Table S2. Relative energy barrier in [kcal/mol] as a result of the S1-C2-N4-C1 dihedral angle rotation of the AZM molecule. The energy estimation was carried out on the basis of the relaxed scan results.

Level of theory	Relative energy
**ωB97XD/aug-cc-pVDZ**	9.907
**M05/aug-cc-pVDZ**	8.978
**M05-D3(0)/aug-cc-pVDZ**	8.959
**M05-2X/aug-cc-pVDZ**	10.788
**M05-2X-D3(0)/aug-cc-pVDZ**	10.804
**M06/aug-cc-pVDZ**	9.827
**M06-D3(0)/aug-cc-pVDZ**	9.837
**M06-2X/aug-cc-pVDZ**	10.088
**M06-2X-D3(0)/aug-cc-pVDZ**	10.101
**M08-HX/aug-cc-pVDZ**	10.665
**MP2/aug-cc-pVDZ**	9.638
**CCSD/aug-cc-pVDZ//MP2/aug-cc-pVDZ**	9.726
**CCSD(T)/aug-cc-pVTZ//MP2/aug-cc-pVDZ**	9.575

Table S3. Interaction energy (E2) between lone electron pairs on the O1 oxygen atom and antibonding σ*(C3–S1) orbital (kcal/mol) in the studied complexes. The calculations were performed at the M06/aug-cc-pVDZ level of theory.

System	[LP2(O1) → σ*(C3-S1)]	[LP1(O1) → σ*(C3-S1)]
**AZM**	2.93	0.72
**AZM-T**	2.85	0.69
**AZM-TT**	3.06	0.74
**AZM-TTL**	3.56	0.87
**AZM-TTLH**	3.55	0.89

The existence of intramolecular chalcogen bond between the oxygen and sulfur atoms belonging to the same molecule is confirmed by the results of the NBO analysis. And so, in all investigated systems, this bond is present through the overlapping of the orbitals of the lone electron pairs LP1(O1) or LP2(O1) of the oxygen atom with the antibonding σ*(C3-S1) orbital. The interaction energies between the respective orbitals are summarized in Table S3 and the pictorial representation of the NCIs is given in Figure S6. As can be seen in each case, the energy E2 for the interaction [LP2(O1) → σ*(C3-S1)] is about four times greater than the interaction energy calculated for the [LP1(O1) → σ*(C3-S1)] donation. Thus, for example for the AZM-T, these energies are 2.93 and 0.72 kcal/mol, respectively. For this dimer, the orbitals of the donor and acceptor of the electron density involved in the above-mentioned two interactions are shown in Figure S7. As can be seen from the data collected in Table S3, the intermolecular chalcogen bond is present in all the studied complexes. Moreover, in the case of AZM-TTL and AZM-TTLH, the interaction energies are clearly greater than in the case of AZM. It could be explained by the presence of intermolecular hydrogen bonds in which the sulfur atom is involved as a donor of the electron density. Thus, for example, in AZM-TTL, donation is observed from two lone electron pairs of the sulfur atom, LP1(S1) and LP2(S1), to the three antibonding σ*(C-H) orbitals as well as to the Rydberg orbitals of the three nearest hydrogen atoms. The largest interaction energies between the mentioned orbitals have a value of 0.81 kcal/mol. The results of the NBO analysis confirm the fact that in the dimer AZM-TT the sulfur atom is involved not only in the intramolecular chalcogen bond but also in the intermolecular one. In the latter case, the donor is the one of the nitrogen atoms of the TT dipeptide. There is an interaction between the orbital of the lone electron pair of the

nitrogen atom LP(N) and the sulfur Rydberg orbital Ry*(S1). The value of the interaction energy between these orbitals is only 0.12 kcal/mol.

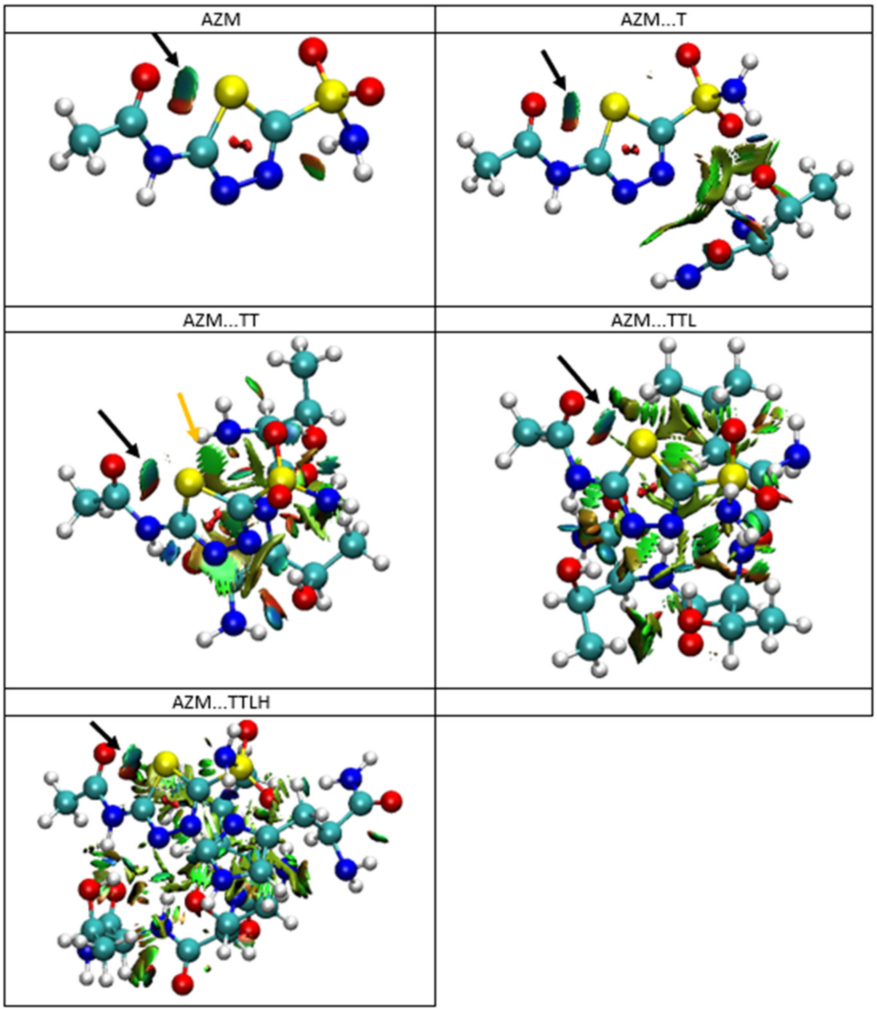

Figure S6. NCI diagrams of the investigated systems. Colors represent various type of interaction: green – interaction of average strength, brown – very weak interactions, red – repulsion. Black arrows point to the green regions corresponding to the intramolecular chalcogen bond (between oxygen and sulfur atoms), while orange ones indicate at intermolecular chalcogen bond (between oxygen and nitrogen atoms).

LP1(O1) →σ*(C3-S1)	LP2(O1) →σ*(C3-S1)
E^2 = 0.72 kcal/mol	E^2 = 2.93 kcal/mol
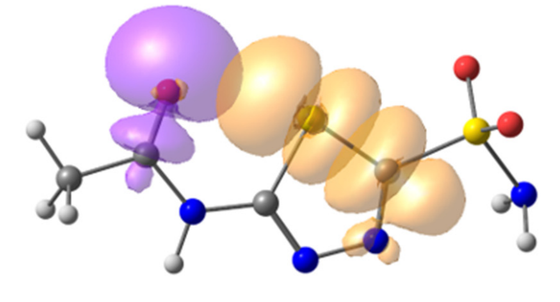	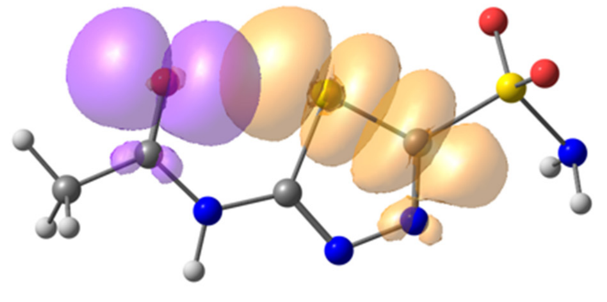

Figure S7. The isosurface (on the 0.03 isovalue) of the interacting orbitals LP1(O1) or LP2(O1) and $\sigma*$(C3-S1) in the AZM molecule. The LP(O1) orbitals are violet-colored, while $\sigma*$(C3-S1) is orange.

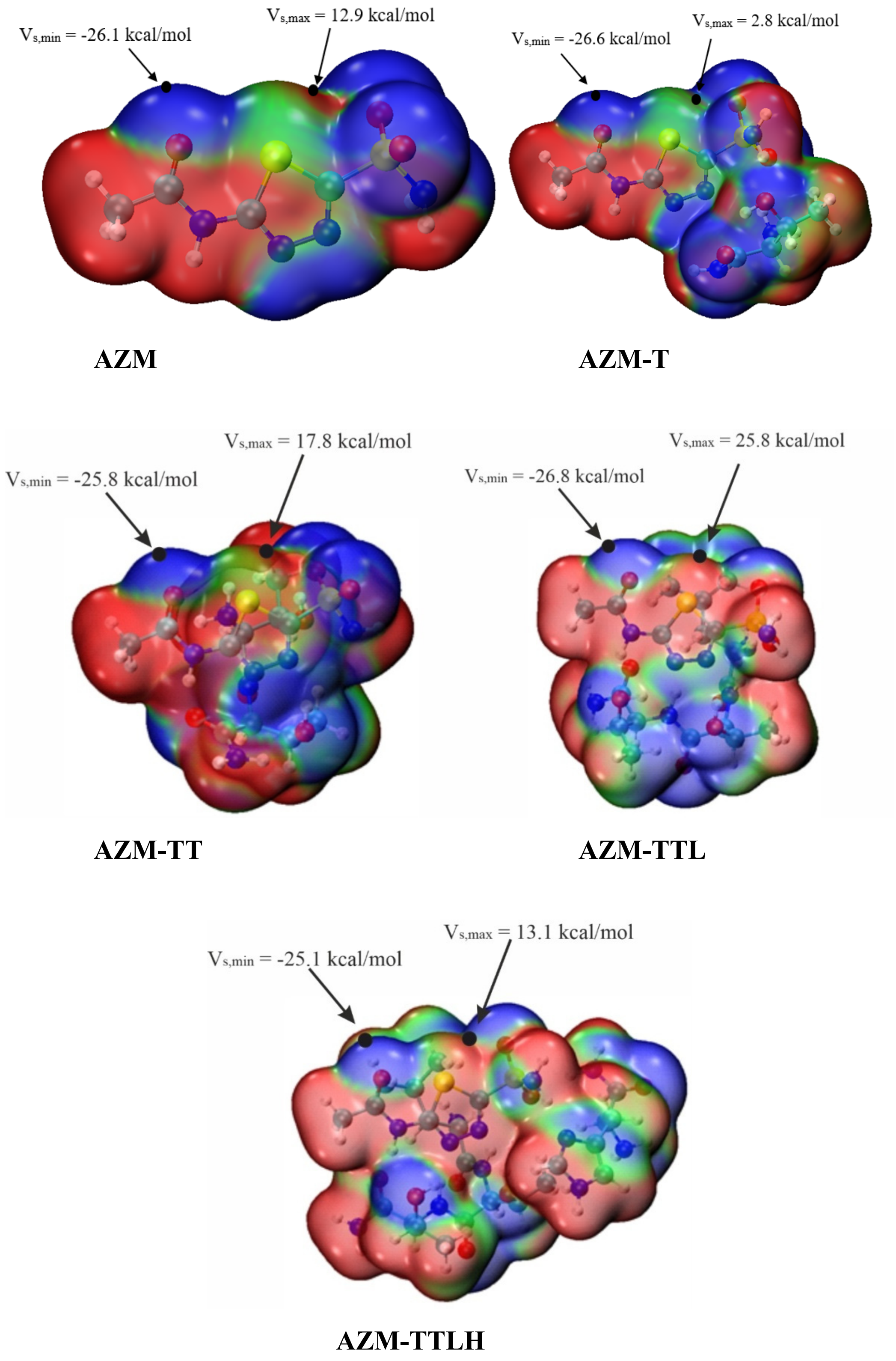

Figure S8. Molecular Electrostatic Potential (MEP) isosurfaces on the 0.001 a.u. isodensity surface computed at the M06/aug-cc-pVDZ level of theory for the AZM molecule and its complexes with amino acids. Scale: from -0.01 (blue) to 0.01 a.u. (red). The two extremes are marked with black dots

Figure S8 shows the electrostatic potential surfaces for the isolated system (AZM) as well as for its complexes with amino acids. Table S4 collects the values of $V_{s,max}$ and $V_{s,min}$ (for all five studied complexes) located near the corresponding atoms involved in the intramolecular chalcogen bond. From the values for the isolated system (AZM) it can be assumed that the π-hole potential on the S atom, which is not involved in the intramolecular chalcogen bond, would be about 13 kcal/mol while the $V_{s,min}$ value on the oxygen atom in the absence of this chalcogen bond would be about -26 kcal/mol. It can be seen from Table S4 that the formation of complexes affects only slightly the $V_{s,min}$ on the oxygen atom which varies from -25 to -27 kcal/mol. In the case of $V_{s,max}$ located in the vicinity of the sulfur atom the values of the π-hole strength are clearly larger and vary from about 3 to 26 kcal/mol. Higher values of potential than in the monomer or the AZM-T complexes result from the presence of hydrogen atoms near the sulfur center.

Table S4. Values of selected extremes on 0.001 a.u. isodensity MEP surface calculated at the M06/aug-cc-pVDZ level of theory for the studied AZM molecule and its complexes with amino acids.

System	$V_{s,min}$	$V_{s,max}$
AZM	-26.1	12.9
AZM-T	-26.6	2.8
AZM-TT	-25.8	17.8
AZM-TTL	-26.8	25.8
AZM-TTLH	-25.1	13.1

Table S5. AIM-derived properties at BCPs of intermolecular non-covalent interactions of equilibrium structures. $E1_{HB}$ and $E2_{HB}$ are intermolecular hydrogen bond energies [kcal/mol] based on Espinosa and Vener equation. Electron density, ρ_{BCP}, is given in $e*a_0^{-3}$ and its Laplacian, $\nabla^2\rho_{BCP}$, in $e*a_0^{-5}$. V_{CP} and G_{CP} denote potential and kinetic energy density at the BCPs respectively.

System	BCP	Amino acid	ρ	$\nabla^2\rho$	V_{CP}	G_{CP}	$E1_{HB}$	$E2_{HB}$
AZM-T	H3A•••O-H	T199	0.0309	0.1145	-0.0232	0.0259	7.2881	6.9802
	O2A•••H-NH	T199	0.0149	0.0463	-0.0109	0.0112	3.4096	3.0204
AZM-TT	H4•••O=C	T200	0.0270	0.0955	-0.0197	0.0218	6.1886	5.8691
	O2A•••H-O	T200	0.0236	0.0788	-0.0174	0.0186	5.4587	4.9946
	N2•••H-O	T200	0.0209	0.0660	-0.0128	0.0147	4.0171	3.9439
	N1•••H-N	T200	0.0132	0.0403	-0.0091	0.0096	2.8515	2.5779
	H3A•••O-H	T199	0.0194	0.0662	-0.0147	0.0156	4.6083	4.2048
AZM-TTL	N2•••H-O	T199	0.0234	0.0759	-0.0144	0.0167	4.5114	4.4881
	N1•••H-O	T200	0.0227	0.0698	-0.0150	0.0162	4.6986	4.3637
	H4•••O-H	T200	0.0246	0.0801	-0.0175	0.0188	5.5026	5.0576
	H4•••O=C	T200	0.0101	0.0353	-0.0073	0.0080	2.2743	2.1620
	O2A•••H-N	T199	0.0103	0.0321	-0.0072	0.0076	2.2569	2.0468
AZM-TTLH	H4•••O=C	T200	0.0269	0.0974	-0.0195	0.0219	6.1156	5.9005
	N1•••H-O	T200	0.0186	0.0537	-0.0119	0.0127	3.7391	3.4109
	N1•••H-N	T200	0.0148	0.0407	-0.0093	0.0097	2.9179	2.6222
	N2•••H-O	T199	0.0210	0.0648	-0.0129	0.0145	4.0340	3.9103
	N2•••H-N	T199	0.0133	0.0379	-0.0087	0.0091	2.7232	2.4420
	O2A•••H-NH	H96	0.0182	0.0584	-0.0128	0.0137	4.0158	3.6884
	H3A•••ND	H96	0.0408	0.1197	-0.0286	0.0293	8.9694	7.8764

Table S6. AIM-derived properties at BCPs of the investigated systems (excluding S1…O1 chalcogen bond) taken from the CPMD runs. The $E1_{HB}$ and $E2_{HB}$ are intermolecular hydrogen bond energies [kcal/mol] based on Espinosa and Vener equation. Electron density, ρ_{BCP}, is given in $e^*a_0^{-3}$ and its Laplacian, $\nabla^2\rho_{BCP}$, in $e^*a_0^{-5}$. V_{CP} and G_{CP} denote the potential and kinetic energy density at the BCPs respectively.

System	BCP	Amino acid	ρ	$\nabla^2\rho$	V_{CP}	G_{CP}	$E1_{HB}$	$E2_{HB}$
AZM-T(s)[a]	N2•••H-O	T199	0.0434	0.1401	-0.0337	0.0343	10.5819	9.2544
	H3B•••O-H	T199	0.0244	0.0747	-0.0169	0.0178	5.2859	4.7815
	O2B•••H-NH	T199	0.0128	0.0396	-0.0090	0.0094	2.8228	2.5436
AZM-T(l)[a]	O1•••H-NH	T199	0.0117	0.0378	-0.0088	0.0091	2.7474	2.4518
AZM-TT(s)[a]	H4•••O=C	T200	0.0303	0.1008	-0.0210	0.0231	6.6017	6.2228
	N1•••H-O	T200	0.0229	0.0696	-0.0140	0.0157	4.4132	4.2347
	N1•••H-N	T199	0.0148	0.0428	-0.0098	0.0102	3.0628	2.7545
AZM-TT(l)[a]	N2•••H-O	T200	0.0092	0.0308	-0.0065	0.0071	2.0282	1.9077
	N1•••O=C	T200	0.0140	0.0505	-0.0098	0.0112	3.0878	3.0242
	H4•••O=C	T200	0.0455	0.1718	-0.0397	0.0413	12.4511	11.1237
AZM-TTL(s)[a]	H4•••O=C	T200	0.0234	0.0759	-0.0144	0.0167	4.5114	4.4881
	H4•••O-H	T200	0.0227	0.0698	-0.0150	0.0162	4.6986	4.3637
	N2•••H-O	T199	0.0246	0.0802	-0.1754	0.0188	5.5026	5.0576
	N1•••H-N	T200	0.0282	0.0950	-0.0206	0.0222	6.4573	5.9673
	N1•••H-O	T200	0.0151	0.0411	-0.0095	0.0099	2.9693	2.6589
AZM-TTL(l)[a]	N2•••H-N	T199	0.0401	0.1375	-0.0302	0.0323	9.4705	8.6891
	H4•••O-H	T200	0.0139	0.0407	-0.0092	0.0097	2.8921	2.6094
	N1•••H-N	T200	0.0125	0.0352	-0.0081	0.0084	2.5292	2.2687
	H3A•••O-H	T199	0.0203	0.0608	-0.0129	0.0141	4.0603	3.7868
AZM-TTLH(s)[a]	H4•••O-H	T200	0.0329	0.1170	-0.0245	0.0269	7.6774	7.2390
	N1•••H-N	T200	0.0157	0.0391	-0.0089	0.0093	2.7920	2.5151
	N1•••H-O	T199	0.0129	0.0416	-0.0089	0.0097	2.8038	2.6033
	N2•••H-N	T199	0.0088	0.0264	-0.0055	0.0060	1.7112	1.6218
	H3A•••ND	H96	0.0190	0.0497	-0.0110	0.0117	3.4491	3.1532
	O2A•••H-N	H96	0.0096	0.0309	-0.0069	0.0073	2.1662	1.9681
AZM-TTLH(l)[a]	H4•••O-H	T200	0.0370	0.1390	-0.0291	0.0319	9.1334	8.5882
	N1•••H-N	T200	0.0230	0.0699	-0.0136	0.0156	4.2817	4.1878
	N2•••H-O	T199	0.0209	0.0616	-0.0135	0.0144	4.2289	3.8857
	N2•••N-H	T199	0.0093	0.0275	-0.0056	0.0062	1.7569	1.6776
	H3A•••ND	H96	0.0378	0.1210	-0.0264	0.0283	8.2980	7.6150
	O2A•••H-N	H96	0.0169	0.0542	-0.0123	0.0129	3.8661	3.4824

[a]AZM-T(s) and AZM-T(l) denote conformations where the S1•••O1 chalcogen bond was the shortest and the longest, respectively.

Tables S5 and S6 gather physical quantities derived from the AIM method. The results were obtained for intermolecular interactions of the optimized structures (static model) and from the CPMD run. The AIM analysis was employed to obtain not only a qualitative, but also a quantitative picture of the intermolecular interactions in the studied complexes. It was mentioned, when the metric parameters of S1•••O2A and S1•••O2B have been discussed, that the system propensity to vary the S1-C3-S2-O2A torsional angle is associated with the chemical equivalence of both O2A and O2B and it is highly dependent on the intramolecular bonds formation (as the results of PIMD proved). The data from Table S6 have shown that the S1-C3-S2-O2A variation have stopped, when the O2A (O2B) and H3A (H3B) have started to get involved in the stronger intermolecular hydrogen bonds – it can be easily seen, when one compares the sum of O2A and H3N interaction energies of AZM-TTLH with the smaller complexes. Other intermolecular non-covalent interactions, besides the abundant hydrogen bonds, were also detected in the examined systems: in the case of AZM-TT(l) complex the pnicogen bond N1•••O=C was present, whereas for the AZM-TTLH(l) the weak pnicogen interaction N2•••N-H was observed.

Table S7. AIM-derived properties at BCPs of the investigated complexes (excluding the S1•••O1 chalcogen bond). The number next to the system designation describes the rotation along the S1-C2-N4-C1 torsional angle. Relaxed scan was performed at the M06/aug-cc-pVDZ level of theory with S1-C2-N4-C1 torsional angle rotation as a variable. E1 and E2 are hydrogen bond energies computed based on Espinosa and Vener equation. The dimensions of given quantities are as follows: ρ_{BCP} is given in e*a_0^{-3} , its Laplacian, $\nabla^2\rho_{BCP}$, in e*a_0^{-5}, whereas V_{CP} and G_{CP} denote the potential and kinetic energy density at the BCPs respectively.

System	BCP	Amino	ρ	$\nabla^2\rho$	V_{CP}	G_{CP}	E1	E2
AZM-T-90	O2A•••H-N	T199	0.0195	0.0647	-0.0137	0.0149	4.3029	4.0226
	H3A•••O-H	T199	0.0325	0.1203	-0.0249	0.0275	7.8222	7.4049
AZM-T-180	N1•••O1	--	0.0128	0.0456	-0.0094	0.0104	2.9507	2.7986
	N2•••H-N	T199	0.0118	0.0338	-0.0076	0.0080	2.3891	2.1624
	N1•••NH2	T199	0.0150	0.0546	-0.0092	0.0114	2.8861	3.0754
	O1•••H-N	T199	0.0174	0.0570	-0.0121	0.0132	3.8003	3.5468
	H3A•••O=C	T199	0.0305	0.1141	-0.0232	0.0259	7.2875	6.9656
AZM-T-270	H3A•••O-H	T199	0.0324	0.1220	-0.0253	0.0279	7.9282	7.5075
	O2A•••H-N	T199	0.0230	0.0821	0.0185	0.0185	5.1544	4.9747
	O1•••H-N	T199	0.0223	0.0760	-0.0156	0.0173	4.8968	4.6588
AZM-TT-90	O2A•••H-O	T199	0.0220	0.0727	-0.0161	0.0171	5.0406	4.6084
	N1•••H-N	T200	0.0140	0.0456	-0.0099	0.0106	3.1033	2.8665
	N2•••H-O	T200	0.0213	0.0661	-0.0131	0.0148	4.1066	3.9864
	H3A•••O-H	T199	0.0238	0.0838	-0.0176	0.0193	5.5259	5.1915
	O1•••H-N	T199	0.0194	0.0659	-0.0142	0.0153	4.4498	4.1248
AZM-TT-180	N1•••O1	--	0.0131	0.0493	-0.0096	0.0110	3.0261	2.9570
	N2•••H-O	T199	0.0248	0.0817	-0.0157	0.0181	4.9205	4.8591
	O2A•••H-O	T199	0.0218	0.0697	-0.0167	0.0170	5.2232	4.5851
	H3A•••O-H	T199	0.0211	0.0716	-0.0160	0.0169	5.0048	4.5561
	N1•••H-N	T200	0.0153	0.0470	-0.0099	0.0108	3.0995	2.9104
AZM-TT-270	H4•••O=C	T200	0.0235	0.0760	-0.0173	0.0182	5.4384	4.8889
	O2A•••H-O	T199	0.0222	0.0760	-0.0166	0.0178	5.2199	4.7954
	N3•••H-O	T199	0.0206	0.0610	-0.0115	0.0134	3.5919	3.5925

Table S7 (Continuation). AIM-derived properties at BCPs of the investigated complexes (excluding the S1•••O1 chalcogen bond). The number next to the system designation describes the rotation along the S1-C2-N4-C1 torsional angle. Relaxed scan was performed at the M06/aug-cc-pVDZ level of theory with S1-C2-N4-C1 torsional angle rotation as a variable. E1 and E2 are hydrogen bond energies computed based on Espinosa and Vener equation. The dimensions of given quantities are as follows: ρ_{BCP} is given in $e{*}a_0^{-3}$, its Laplacian, $\nabla^2\rho_{BCP}$, in $e{*}a_0^{-5}$, whereas V_{CP} and G_{CP} denote the potential and kinetic energy density at the BCPs respectively.

System	BCP	Amino	ρ	$\nabla^2\rho$	V_{CP}	G_{CP}	E1	E2
AZM-TTL-90	N1•••H-N	T200	0.0138	0.0372	-0.0085	0.0089	2.6730	2.3992
	N2•••H-N	T199	0.0211	0.0652	-0.0133	0.0148	4.1676	3.9802
	N2•••H-O	T199	0.0132	0.0384	-0.0088	0.0092	2.7645	2.4780
	H4•••O-H	T200	0.0259	0.0853	-0.0183	0.0198	5.7537	5.3400
AZM-TTL-180	N1•••H-N	T200	0.0130	0.0373	-0.0077	0.0085	2.4219	2.2931
	N4•••O-H	T200	0.0116	0.0406	-0.008	0.0092	2.5694	2.4675
	N1•••O1	--	0.0131	0.0488	-0.0096	0.0109	3.0181	2.9374
AZM-TTL-270	N3•••H-O	T199	0.0186	0.0526	-0.0103	0.0117	3.2406	3.1605
	O1•••H-N	T199	0.0194	0.0723	-0.0151	0.0166	4.7336	4.4637
	O1•••H-N	T200	0.0101	0.0321	-0.0074	0.0077	2.3294	2.0806

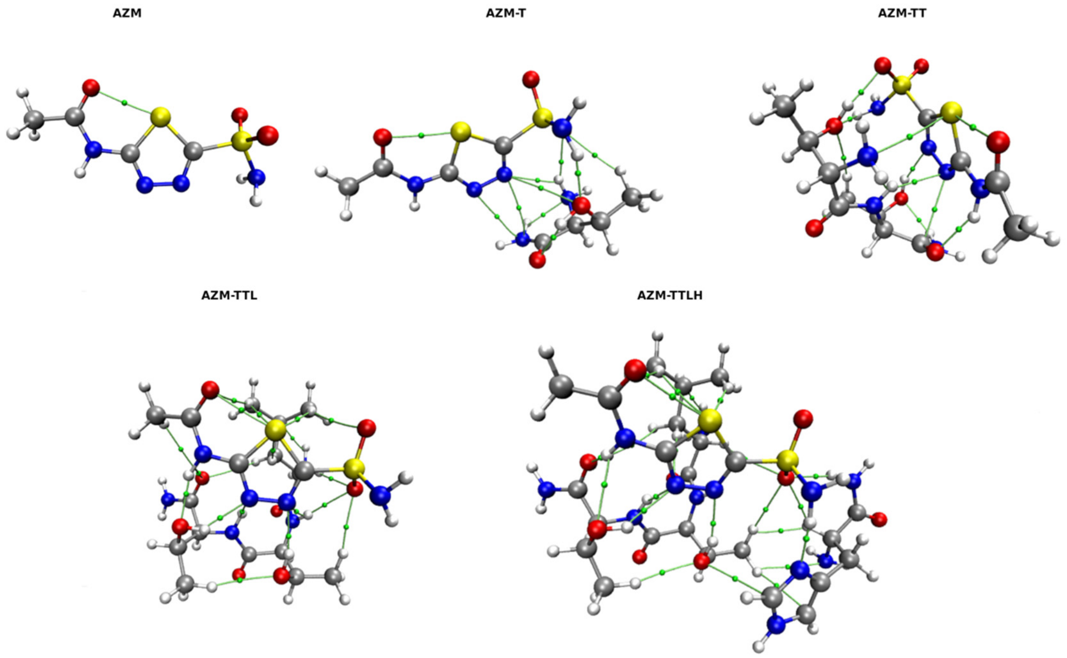

Figure S9. Atoms in Molecules (AIM) molecular graphs of the AZM molecule and its complexes with amino acids. The simulations were performed at the M06/aug-cc-pVDZ level of theory. Only BCPs of non-covalent interactions are presented and marked as small green spheres along bond paths.

Table S8. Decomposition of the interaction energy of complexes into Pauli repulsion (E_{pauli}), electrostatic (E_{elec}), orbital interaction (E_{oi}) and dispersion (E_{disp}) components. All energies in kcal/mol.

System	Level of theory	E_{Pauli}	E_{elec}	%[a]	E_{oi}	%[a]	E_{disp}	%[a]	E_{int}
**AZM-T**	**M06/TZ2P**	15.11	-21.8	69	-9.87	31	--	--	-16.56
**AZM-T**	**PBE0-D3(0)/TZ2P**	21.86	-22.04	57	-10.84	28	-5.97	15	-16.99
**AZM-TT**	**M06/TZ2P**	27.64	-38.06	66	-19.33	34	--	--	-29.76
**AZM-TT**	**PBE0-D3(0)/TZ2P**	42.06	-38.93	54	-21.5	30	-11.23	16	-29.60
**AZM-TTL**	**M06/TZ2P**	25.74	-38.8	65	-20.5	35	--	--	-33.56
**AZM-TTL**	**PBE0-D3(0)/TZ2P**	42.63	-40.18	52	-22.77	29	-15.01	19	-35.34

[a]percentage contribution to total attractive interactions (E_{elec}+E_{oi}+E_{disp})

A decomposition of the interaction energy (E_{int}) into its components is given in Table S8. Interaction energy decomposition calculations were performed by using two different DFT functionals. One, M06, is as used in the whole paper and the other (PBE0-D3) with added Grimme dispersion correction. The interaction energies calculated using the various functionals are given in the last column of this table. As can be seen the values of the interaction energies calculated using different functionals are close to each other. The differences do not reach more than 6%. According to the data given in Table S8, the electrostatic attraction energy is almost 70% of the total attraction energy. The remainder of this energy is accounted for by the E_{oi} component. The contribution of the dispersion energy calculated at the PBE0-D3/TZ2P level ranges from 15% to 19% for system 2 and 4, respectively. However, we cannot infer more knowledge about the intramolecular chalcogen bond from these data, since the interaction energy covers the intermolecular factors and does not take into account the intramolecular components.

References:

[1] A. J. A. Price, K. R. Bryenton, E. R. Johnson, Requirements for an accurate dispersion-corrected density functional, *J. Chem. Phys.* **2021**, 154, 230902. doi:10.1063/5.0050993.

[2] M. Michalczyk, W. Zierkiewicz, R. Wysokiński and S. Scheiner, Theoretical Studies of IR and NMR Spectral Changes Induced by Sigma-Hole Hydrogen, Halogen, Chalcogen, Pnicogen, and Tetrel Bonds in a Model Protein Environment, *Molecules* **2019**, 24, 3329. doi:10.3390/molecules24183329.

[3] C. Genis, K. H. Sippel, N. Case, W. Cao, B. Sankara Avvaru, L. J. Tartaglia, L. Govindasamy, C. Tu, M. Agbandje-McKenna, D. N. Silverman, C. J. Rosser, R. McKenna, Design of a Carbonic Anhydrase IX active-site mimic to screen inhibitors for possible anticancer properties. *Biochemistry* **2009**, 48, 1322-1331. doi: 10.1021/bi802035f.
